# Evaluation of YOLOv7-v13 models for multi-class small insect pest detection using the five-pest dataset

**DOI:** 10.1038/s41598-026-52108-3

**Published:** 2026-05-20

**Authors:** Ayesha Hakim, M. Habib Ur-Rahman, Ali Hamza, Munir P. Hoffmann, Vakhtang Shelia, Muhammad Owais, Nimra Khan, Muhammad Saim Ibtesam, Muhammad Rashid, Reimund P. Roetter

**Affiliations:** 1 School of Electrical Engineering and Computer Science (SEECS), National University of Sciences and Technology (NUST), Islamabad, Pakistan; 2 Institute of Computing, MNS University of Agriculture, Multan, Pakistan; 3 Tropical Plant Production and Agricultural Systems Modelling (TROPAGS), Department of Crop Sciences, University of Göttingen, Grisebachstraße 6, 37077 Göttingen, Germany; 4 Department of Agricultural and Biological Engineering, University of Florida, Gainesville, FL 32611 USA; 5 Institute of Plant Protection, MNS University of Agriculture, Multan, Pakistan; 6 Centre of Biodiversity and Sustainable Land Use (CBL), University of Göttingen, Büsgenweg 1, 37077 Göttingen, Germany; 7 Agvolution , GmbH, Geismar Landstrasse 11, 37083, Göttingen, Germany

**Keywords:** Single stage detector, Deep learning, PGI, GELAN, CNN, Maize, Mango, Cotton, Model ensemble, Small-object detection, Hyperparameter optimization, Ecology, Ecology, Zoology

## Abstract

Pest infestation affects global agriculture, causing massive crop yield losses, ecosystem degradation, and negative economic impacts. High accuracy and efficiency in detecting small insect pests are crucial to avoid pesticide overuse and biodiversity loss. The objective of this research was to evaluate state-of-the-art object detection models (YOLOv7-v13) for multi-pest identification, focusing on small insect (< 5% image area) in mango (fruit flies), maize (fall armyworm), and cotton crops (pink bollworm) for effective decision-making. We developed the Five-Pest dataset comprising 17,251 images captured by IoT-based smart traps and 194,050 pest instances of five economically significant species (three fruit fly species, fall armyworm, and pink bollworm). Images were annotated via Roboflow, then augmented and smoothed ($\varepsilon = 0.1$). YOLO models were trained under identical hyperparameters (40 epochs, batch 8, IoU = 0.8, max_det = 550). Performance was assessed by mAP@50, precision, recall, F1-score, and ROC-AUC. YOLOv9 achieved the highest mAP@50 (0.929), an average that includes lower precision such as fruit flies, underscoring the model’s robustness across varied pest types. YOLOv9 was followed by YOLOv8 (0.924), YOLOv12 (0.922), YOLOv10 (0.921), YOLOv11 (0.909), and YOLOv13 (0.909), while YOLOv7 achieved mAP@50 of 0.899 for the Five-Pest dataset. YOLOv12 demonstrated comparable performance to YOLOv8 and YOLOv10 with stable precision and recall, whereas YOLOv13 achieved competitive detection performance but required substantially higher training time, indicating the accuracy-computational cost trade-off in later YOLO generations. All models detected fall armyworm with very high accuracy (mAP 0.96–0.99), while fruit flies (especially *B. zonata*) remained comparatively more challenging. Pink bollworm recognition remained consistently strong across all variants (mAP 0.91–0.97). To improve per-class detection robustness, an ensemble model averaging YOLOv7-YOLOv13 predictions showing enhanced consistency but requiring more computation. Here we identified the strengths and limitations of YOLO variants for small-insect detection, guiding the selection of models to help reducing pesticide use and enhancing environmental protection in precision agriculture.

**Supplementary Information:**

The online version contains supplementary material available at 10.1038/s41598-026-52108-3.

## Introduction

Current agricultural production systems face increasing vulnerability due to global climate and environmental change^1–7^. Insect pest infestation aggravates these threats, causing significant yield losses of between 40 and 80%, and declining food quality, depending on environmental conditions and genotypic variation^8^. Among these pests, three species of fruit flies (*Bactrocera cucurbitae*, *B. zonata*, *B. dorsalis*), pink bollworm (*Pectinophora gossypiella*) in cotton, and fall armyworm (*Spodoptera frugiperda*) in maize are especially damaging, impacting both domestic production and export potential^3,8–11^. Fruit flies are one of the most harmful insect pests that affect crops through direct damage to the fruits. The fruit fly infestation is a significant hindrance to exporting fresh fruits and vegetables to international markets^3,12,13^. There is a critical need to develop smart technologies for pest management and sustainable crop production to tackle the negative impacts of environmental stress and meet growing food demands and nutritional ﻿quality^14–16^, 39,40, 43] .

Early and accurate detection of these insects is critical for reducing indiscriminate pesticide applications and preserving biodiversity. Their tiny size and often dense grouping on plant surfaces challenge traditional visional systems and require specialized detection strategies. In this study, we define small insects as those occupying no more than 5% of the image area (roughly under 32 × 32 pixels at 640 × 640 resolution). Advances in deep learning have transformed object detection, i.e. moving beyond single-category classification^17^ towards robust multi-object localization. Single-stage detectors like YOLO process the entire image in one network pass, offering high inference speed at the cost of some localization precision, an acceptable trade-off for real-time agricultural monitoring^31^. Convolutional neural networks and transformer-based models continue to push boundaries in detection accuracy and efficiency, yet most pest-detection studies focus on single-species or general benchmarks.

Recent studies have applied various YOLO architectures to pest detection with notable success^18^. compared YOLOv7-tiny and YOLOv8n for olive fruit flies, finding v7-tiny more accurate^19^. enhanced YOLOv8 with a DCF module on the IP102 dataset of thistle caterpillars, red beetles, and citrus psylla, achieving 84.7% mAP outperforming YOLOv4, YOLOv3, and YOLOv6 (71.5%, 58.1%, and 76.4% respectively)^20^. presented an enhanced YOLOX for forest pest detection, surpassing YOLOv3, Faster R-CNN, and the original YOLOX model^21^. introduced Pest-YOLO for dense, multi-class small-insect detection on a 20 k-image dataset with over 190 k instances. Similarly^22^, combined SAHI with YOLOv8 for tea-crop micro-pest recognition, and^23^ developed YOLO-Pest using the CAC3 module for improved feature aggregation^24^. proposed PestLite, a YOLO-based method for broad field-crop pest detection, demonstrating the continued relevance of YOLO frameworks in diverse agricultural contexts. Recent work by^25^ has further highlighted the importance of task-oriented enhancements in YOLO frameworks combined with domain-specific datasets for improving small-object detection under complex environmental conditions. Despite these advances, there is a lack of unified, multi-species benchmarks using the very latest YOLO variants, v7 through v13, on a dataset tailored to small-insect detection. The existing studies rarely address dense insect clusters under variable lighting and backgrounds, nor do they provide clear guidance on real-time deployment constraints in resource-limited agricultural settings.

The current study makes four key contributions to advance research in this field: First, we present the Five-Pest dataset, a new benchmark of 17,251 high-resolution IoT-trap images and ~ 194,050 labeled instances covering five key pest species in mango, maize, and cotton fields. Second, we introduce specialized training strategies, including tailored data augmentations and label-smoothing techniques, to improve detection of small, densely clustered insects. Third, we systematically compare the latest YOLO versions (v7-v13) under uniform conditions, evaluating detection accuracy, training stability, and computational performance. Finally, and fourth, we demonstrate that a simple weighted ensemble of these models yields further gains in detection accuracy, and we outline practical real-time deployment targets for edge devices and GPUs. By focusing on real-world, multi-pest scenarios, our work bridges algorithmic innovation and the solutions for pest management in agriculture.

## Materials and methods

### Methodological framework and dataset

**Fig. 1:**
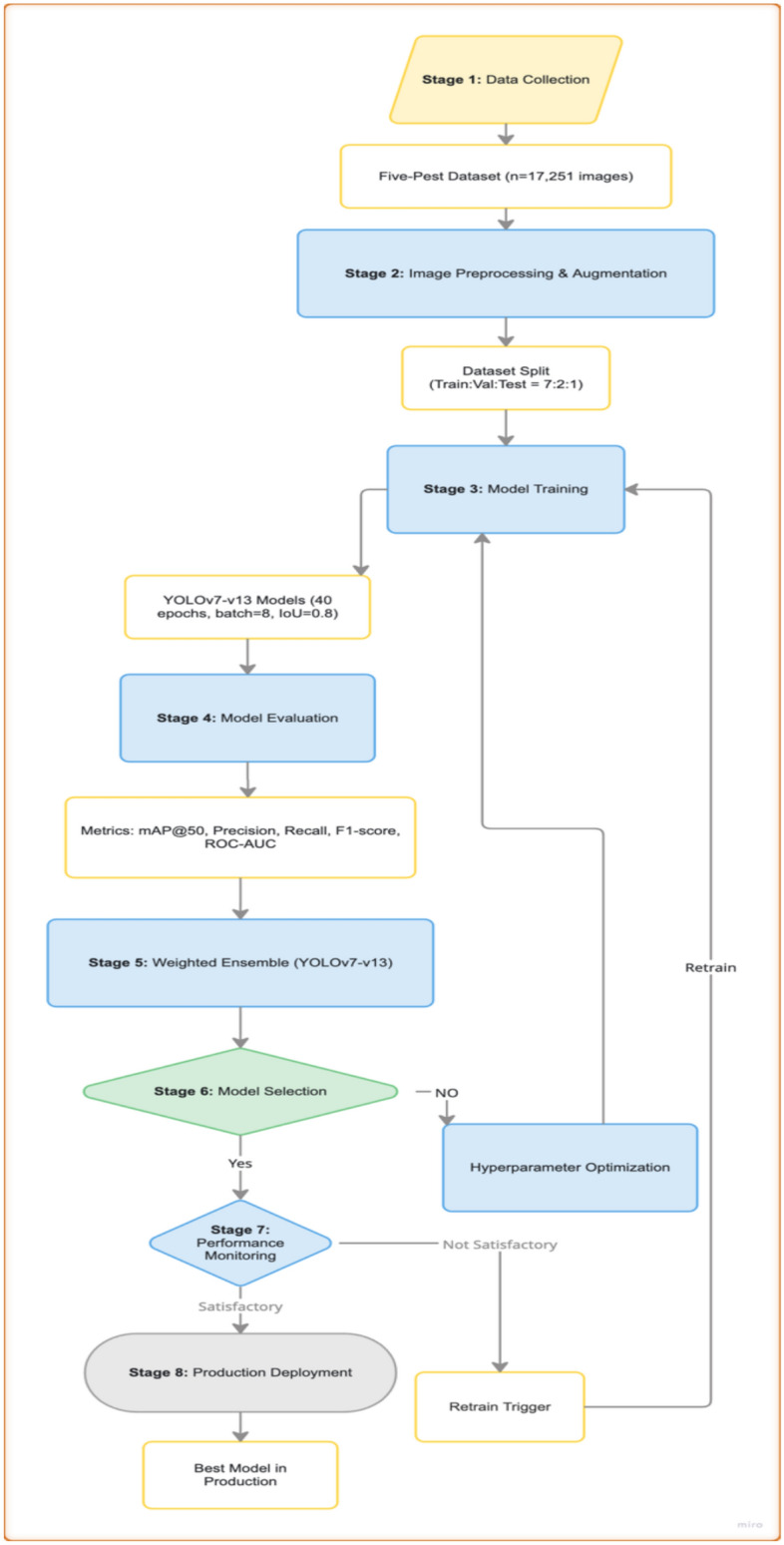
Workflow diagram depicting an end-to-end automated pipeline for pest image collection, pre-processing, labelling, and training multiple YOLO variants. A performance monitoring and feedback loop ensures continuous improvement by triggering retraining when performance degradation is detected.

The workflow diagram depicts an end-to-end automated pipeline for pest image collection, pre-processing, labeling, and training multiple YOLO models (Fig. 1). The process includes hyperparameter optimization, model evaluation (ROC, mAP, F1-score), and model averaging for selection. A performance monitoring and feedback loop ensures continuous improvement by triggering retraining if necessary, leading to the deployment of the best-performing (multi-member) model ensemble. To analyze and compare the selected object detection algorithms, we collected a dataset of five pests, including three species of fruit flies, fall armyworm moths, and pink bollworm moths, using IoT-based smart traps^26^ installed in mango orchards, maize and cotton crop fields, as well as from insect rearing labs.

Different resolutions of cameras captured the images, but most of the images were captured from the smart trap using an ov5640 camera with a resolution of 1920 × 1080. We collected a total of 17,251 images from different agricultural fields using smart traps and insect-rearing labs. The images were collected over a 14-month period from March 2023 to April 2024 across different agroecological zones in South Punjab, Pakistan. This extended collection timeframe ensured coverage of varying seasonal conditions, light variations, pest life cycles, and crop stages, thereby enhancing the dataset’s diversity and robustness for real-world pest detection.

**Fig. 2:**
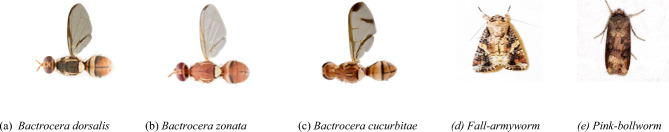
The five selected insect pests included in the dataset for model testing.

Figure 2 shows the close-ups of five insect pests (fruit flies, fall armyworm and pink bollworm moths) whose images are tested in the dataset. To annotate the dataset, we used the roboflow tool^27^ powered by Ultralytics to precisely create bounding boxes around each instance of an image. Each bounding box represents one of the five selected pests, serving to make up the five classes of the dataset: cucurbitae, dorsalis, fall-armyworm, pink-bollworm, and zonata (Fig. 2). After labeling the images, we split the dataset into training, validation, and testing with a ratio of 7:2:1, respectively.

### Data augmentation

Data augmentation is a crucial step in training any deep learning model; it not only increases the size of data, but also increases the generalization of the model. To make this dataset more robust, we applied different augmentation techniques on the training set of datasets, as listed in Table 1. All augmentations were applied online during training (not offline dataset duplication) and were restricted to the training split only after performing the train/validation/test split, to avoid leakage. The augmentation sequence followed a fixed order: (i) geometric transforms (random rotation/shear/scale/translation within the ranges reported in Table 1), followed by (ii) photometric transforms (brightness/contrast and color jitter within Table 1 ranges), and then (iii) optional regularization transforms (blur/noise/cutout where applicable). For geometric transforms, bounding boxes were transformed using the same affine mapping as the image; boxes were then clipped to image boundaries, and boxes with negligible remaining visible area were discarded to prevent invalid labels. This ensures that the annotations remain consistent under rotation/shear operations. Augmentation probabilities were applied uniformly per image per epoch (default online policy), ensuring that each epoch exposes the model to diverse yet label-consistent variants.

**Table 1:** Data augmentation techniques applied to the images in the dataset.

Augmentation	Value
90° Rotate	Clockwise, Counter-Clockwise
Blur	Up to 2px
Brightness	Between −25% and + 25%
Exposure	Between −10% and + 10%
Flip	Horizontal, Vertical
Grayscale	5% of total dataset
Hue	Between -30° and + 30°
Noise	Up to 2% of pixels
Rotate	Between −22° and + 22°
Saturation	Between −20% and + 20%
Cutout	10%, 20 count
Shear	± 8° Horizontal, ± 16° Vertical

After applying image augmentation, the training dataset increased by a factor of three (3), which means the previous pre-training dataset of 5,038 was augmented to 15,114 labelled images, resulting in a total dataset of 17,251 images. Here, instances refer to the total number of annotated pest objects across all classes, and a single image may contain multiple pest instances. Therefore, the instance count is higher than the number of images. Table 2 lists the number of instances of each class in the dataset after augmentation. The dataset contains a total of 194,050 instances of 17,251 images. Specifically, there are 40,406 instances of cucurbitae, 45,431 instances of dorsalis, 20,501 instances of fall-armyworm, 47,061 instances of pink-bollworm, and 40,651 instances of zonata.

**Table 2:** Five classes of the dataset with the number of instances in each class.

Classes	Number of Instances
Cucurbitae	40,406
Dorsalis	45,431
Fall-armyworm	20,501
Pink-bollworm	47,061
Zonata	40,651
Total Instances	194,050

To address the class imbalance issue, we employed the label-smoothing technique^28^, which resolves the issue of limited samples and prevents overfitting of the model. Label smoothing replaces the hard target label with smoothed probabilities that facilitate the model to learn from underlying features by introducing a level of uncertainty in the training labels. The formula for label smoothing is as follows:

1 $$Smooth \,\,label= true\,\, label \left(1 - \varepsilon \right)+\frac{\varepsilon }{n}$$

We set the degree of smoothness to $0.1$ which is represented by epsilon ($\varepsilon )$ in Eq. (1), the *true label* represents the predicted class, whereas $n$ represents the total number of classes in the dataset. This value is a commonly used heuristic that provides a balance between regularization and accuracy, ensuring that the model does not assign a probability of 1 to any class, which helps in mitigating overfitting and improves stability during training, especially in datasets with class imbalance or noisy labels, such as insect pest imagery (Müller et al*.,* 2019). Label smoothing improves the generalisation of the model by preventing it from overfitting and exploring alternative predictions during training. This is important for the detection of small objects in image.

Image augmentation techniques were not applied to the validation and testing datasets as they were used to evaluate and validate the performance metrics of trained models, with validation and testing datasets being close to their real-world counterparts. One of the reasons to choose Roboflow tool for annotation is that it provides the dataset in various training formats such as YOLO txt records, tfrecords, and csv. YOLO object detection models use darknet text labeling format, which represents $(x, y)$ coordinates of the center of detected object, as well as height and width of the bounding box. The labeling format in YOLO darknet text labeling format is mentioned in Sup. Fig. 1. This format contains a text file for each image that contains annotation and a numeric representation of the associated label. It also contains a label map that plots numeric identification to the human-readable string.

### Real-time object detection models

We selected seven single stages YOLO variants, v7 through v13, for their proven balance of speed and accuracy in detecting small objects. For all YOLO families evaluated in this study (YOLOv7-YOLOv13), the standard base architecture variant provided by the official implementation was used. All models were trained under the same hyperparameters (40 epochs, batch = 8, IoU = 0.8, max_det = 550) on our Five‑Pest dataset.

**YOLOv7** is a real-time object detection model released in July 2022 that belongs to the YOLO group of models^29^. The architecture of YOLOv7 is a concatenation-based scaling design that preserves model structure when adjusting depth/width for resource constraints. Its integrated Feature Pyramid Network aggregates multi‑scale features, aiding detection of tiny insects. Anchor boxes of varied shapes are placed densely,Non‑Maximum Suppression (NMS) (Sup. Fig. 2) removes overlapping boxes at an IoU threshold of 0.1 to yield clean detections. Its accuracy and speed were higher than those of the previous object detection models. YOLOv7 utilizes a single convolutional neural network (CNN) architecture, such as Darknet and Resnet, for object localization and class probability prediction directly from full images. Please refer Supplementary Methods for more specific details of the model.

**YOLOv8**^30^ was released in January 2023 by Ultralytics^31^. It was built on YOLOv5 backbone with a C2f. (cross‑stage partial) module and a decoupled head, separating classification and localization branches, which enhances small‑object precision. Advanced loss terms (CIoU and Distribution Focal Loss) further improve box regression for minute targets^32^. Please refer Supplementary Methods for more specific details of the model.

**YOLOv9**^33^ was released in February 2024 by the same developers behind YOLOv7. *It* introduced the Generalized Efficient Layer Aggregation Network (GELAN*)* backbone for more efficient layer aggregation and Programmable Gradient Information (PGI), allowing dynamic adjustment of training parameters. This backbone excels at combining fine‑ and coarse‑scale features, crucial for distinguishing small pests against complex backgrounds. Please refer Supplementary Methods for more specific details of the model.

**YOLOv10**, released in May 2024, introduces a novel dual-label assignment strategy, one-to-many and one-to-one prediction heads, balanced by a consistent matching metric m which prioritizes high‑confidence, well‑aligned boxes. This design improves recall in dense insect clusters while maintaining precision.

2 $$m=s. {p}^{a}. IoU(b,{\widehat{b})}^{\beta }$$

Here, “s” represents the confidence score, p denotes the class probability, α and β are scaling parameters, and $IoU(b,{\widehat{b})}^{\beta }$ measures the overlap between the predicted and ground truth bounding boxes. Please refer Supplementary Methods for more specific details of the model.

**YOLOv11**, released in September 2024, features an advanced architecture designed to enhance efficiency and precision in object detection. YOLOv11 enhances multi‑scale fusion using the Spatial Pyramid Pooling—Fast (SPPF) module and Cross Stage Partial Spatial Attention (C2PSA) spatial‑attention blocks. These modules focus the network on critical image regions, boosting detection accuracy for both small and larger pests. For readers seeking full architectural diagrams, module specifications (e.g., C2f., SPPF, C2PSA) and derivations of the consistent matching metric, please refer to Supplementary Methods.

**YOLOV12,** released beginning of 2025^34^, represents a further evolution of the YOLO family, emphasizing attention-driven feature learning and computational efficiency for real-time object detection. Unlike earlier YOLO variants that primarily relied on convolution-based feature extraction, YOLOv12 integrates attention-centric modules to enhance contextual feature representation and improve localization accuracy, particularly for small and visually ambiguous targets. The architecture introduces optimized attention mechanisms and hardware-aware design strategies, enabling faster convergence and improved inference efficiency on modern GPU architectures.

**YOLOv13,** released in June 2025^35^, represents a further refinement of the YOLO series with an emphasis on improving feature representation stability and detection robustness in complex scenes. The architecture incorporates enhanced feature fusion strategies and optimized training dynamics to improve generalization across diverse object scales. Compared with earlier variants, YOLOv13 prioritizes detection reliability and precision under challenging background conditions, which is particularly relevant for small-insect detection in field environments. While offering competitive detection accuracy, the model introduces increased computational complexity due to deeper feature processing and expanded parameterization.

### Hyperparameter tuning

To ensure a fair comparison across all seven models, we trained each using identical hyperparameters (Table 3), including 40 epochs and a batch size of 8. Given 15,155 training samples, this setup yielded 1,895 steps per epoch (40 × 1,895 = 75,800 total steps). In our setting, performance metrics on the validation set stabilized within this schedule, and extending training further was not pursued to avoid introducing unequal tuning across model families. Notably, the objective of this work is comparative benchmarking under a controlled training protocol (same input size, batch size, augmentation policy, and optimizer settings), rather than exhaustive per-model hyperparameter optimization. To compare the seven models/model versions, we used the same hyperparameters as listed in Table 3. Each image was resized to 640 by 640 pixels before training to optimize computational efficiency while retaining sufficient image details for accurate object detection. We used an optimizer called Adam^36^ with a learning rate of $0.001$ for all models. Adam is a popular optimizer for deep learning tasks due to its adaptive learning rate properties, which lead to faster convergence and better performance.

**Table 3:** Hyperparameters tuning for object detection models (YOLOv7-v13).

Parameters	Value
Epochs/Steps	40/75,800
Batch size	8
Img Dim	640
Cache	RAM
Pre-trained	TRUE
IoU (NMS)	0.8
max_det (per image after NMS)	550
label_smoothing	0.1

Label smoothing (ε = 0.1) redistributes 10% probability mass across non-target classes to reduce overconfidence and improve robustness to annotation noise. The learning rate was scheduled to be updated after every epoch to improve model performance. The training loss was monitored throughout training to ensure stable convergence behavior across models. The maximum number of candidate detections was set to 300 to control the number of overlapping predicted regions considered for object detection. This improves the chances of detecting small objects, since each instance in the dataset covers an average $4\%$ of the total image area. The Intersection Over Unions (IoU) value was set to 0.8 for non-maximum suppression (NMS), which is a used to merge highly overlapping predicted bounding boxes during inference (Sup. Fig. 2). This IoU value does not define the true positive matching threshold for evaluation. Model accuracy is reported using mAP@50 (IoU ≥ 0.50) and mAP@50:95, consistent with standard object-detection practice. Therefore, the evaluation does not rely on an IoU = 0.8 matching criterion for true positives, which would indeed be overly strict for small targets. This value was selected considering insect swarming behavior, where insects often appear in close proximity within trap images. The maximum detections per image (max_det) was set to $550,$ meaning at most $550$ predicted bounding boxes are retained per image after NMS filtering.

### Model training

We utilized the Google Colab Pro Notebook for training, providing access to Tesla T4 GPU with 12 GB GPU RAM and 52 GB system RAM. The training data was cached in system RAM before training, which significantly improved training time. The same dataset, data augmentation techniques, and hyperparameters were applied to all three object detectors. The training durations for the models are as follows: YOLOv7 required 17.4 h, YOLOv8 completed training in 13.667 h, YOLOv9 took 21.644 h, YOLOv10 required 9.2 h, YOLOv11 took 10.12 h, YOLOv12 required 21.1 h, and YOLOv13 completed training in 29.2 h. A detailed comparison of the training times for each model is presented in Sup. Fig. 3, while Sup. Fig. 4 shows the output images of each batch during YOLOv7-v13 training, respectively.

### Ensemble modeling

Ensemble modeling is a machine learning approach that combines multiple models to improve overall performance, reduce errors, and enhance robustness^37^. Instead of relying on a single model, ensemble methods can combine information from multiple models to obtain a more stable performance estimate^38^. In this study, weighted model averaging is used as a performance aggregation and model selection strategy rather than prediction-level fusion. Specifically, models with higher mAP@50 scores are assigned greater influence in the aggregated performance score. This approach provides a balanced and performance-driven summary metric across all evaluated models but does not combine bounding-box predictions during inference. Each model’s weight was calculated as a proportion of its mAP@50 relative to the total sum of all models’ mAP@50 values. This ensures that models with higher detection performance contribute more to the final aggregated score.

The weights are calculated by using the following formula:

$${w}_{i}= \frac{{mAP@50}_{i}}{{\sum}_{j=1}^{N}{mAP@50}_{j}}$$

where:

- ${w}_{i}$ is the weight of model $i$,
- ${mAP@50}_{i}$ is the performance score of model $i$,
- $N$ is the total models.

The final weighted aggregated performance score is calculated as follows:

$$Weighted \,\,Average= \sum_{i=1}^{N}{w}_{i} . { mAP@50}_{i}$$

The weights are normalized such that $\sum_{i=1}^{N}{w}_{i}=1$. This weighting scheme emphasizes models with stronger detection performance while still preserving contributions from all evaluated architectures.

### Statistical indices for model evaluation

The performance of each object detection model was compared using precision, recall, mAP, and confusion metrics on validation and testing datasets. Precision is calculated by Eq. (3), where TP is True Positive, and FP is False Positive.

3 $$P=\frac{TP}{TP+FP}$$

Recall is calculated by Eq. (4), whereas FN refers to False Negative.

4 $$R=\frac{TP}{TP+FN}$$

Mean Average Precision (mAP) is obtained by Eq. (5), where* n* refers to the number of classes and AP_t_ refers to the Average Precision of class *t*. AP calculates the approximate area under the P(R) curve. The actual area under the curve, where P(R) is the Precision (P) at Recall (R) is given by the formula (6)

5 $$mAP=\frac{1}{n}{\sum}_{t=1}^{n}{AP}_{t}$$

6 $$AP= {\int}_{0}^{1}P\left(R\right)dR$$

Mean Average Precision (mAP@50) is the measure of how accurately a model can identify objects given that the IoU of the predicted bounding box with the ground truth is at least 50%. A higher mAP@50 score means that it performs well in identifying and locating objects. In addition, ROC curve provides a comprehensive evaluation of model performance across different classification thresholds, addressing limitations of older metrics that rely on a single threshold (e.g., accuracy, precision, recall). By analyzing the trade-off between true positive and false positive rates, the ROC curve enables better inference on model robustness and discriminatory power. This ensures a more nuanced and data-driven approach to performance assessment, aligning with contemporary best practices in machine learning evaluation. By comparing these values, we can figure out which model shows better accuracy and whether each new version of the YOLO family offers improvements over the previous ones.

## Results

**Fig. 3:**
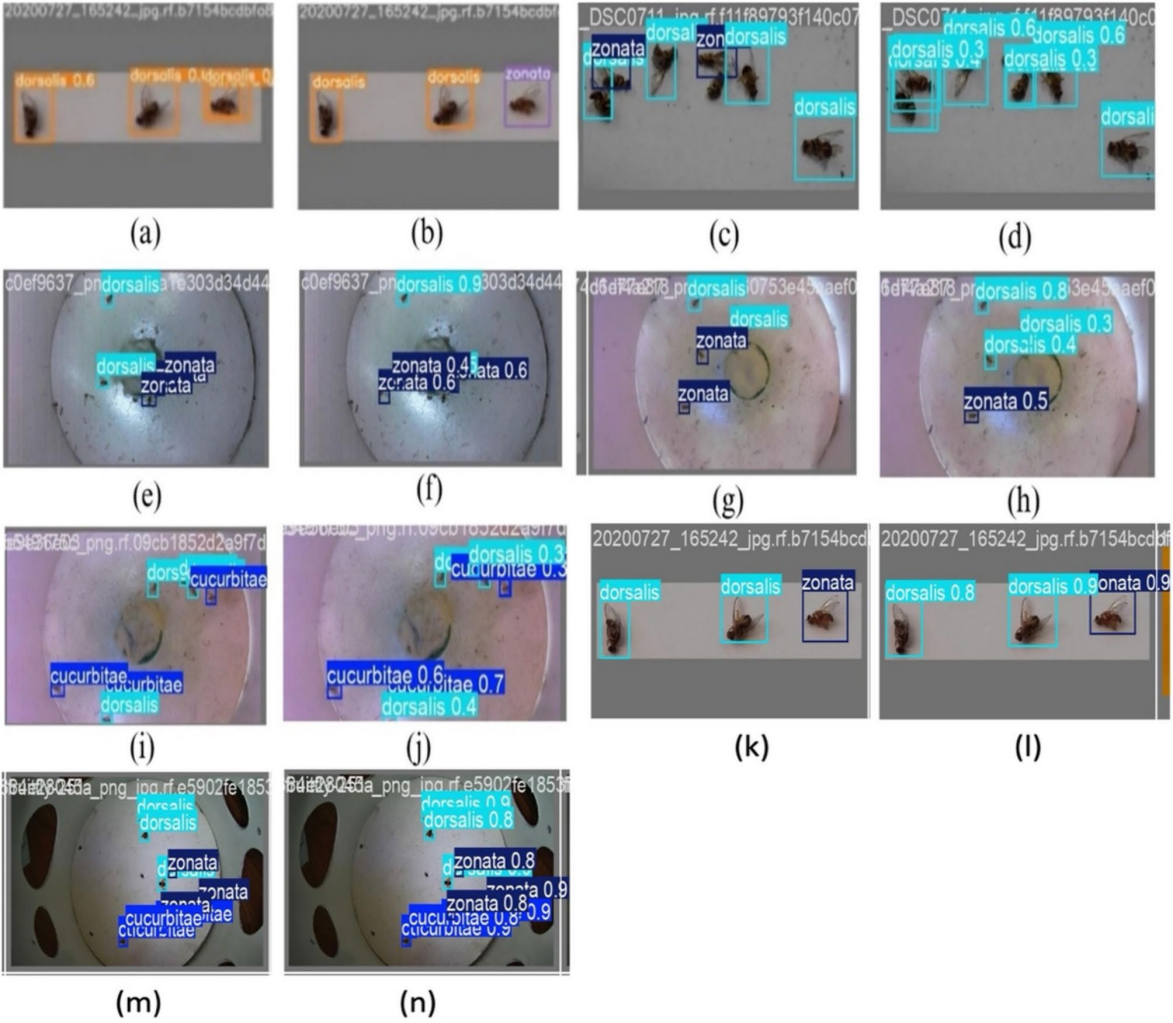
**a**, **c**, **e**, **g**, **i**, **k**, and m representing true labels from validation dataset, while **b**, **d**, **f**, **h**, **j**, **l**, and n representing output results after prediction from YOLO variants (YOLOv7, YOLOv8, YOLOv9, YOLOv10, YOLOv11, YOLOv12, YOLOv13) models, respectively.

**Fig. 4:**
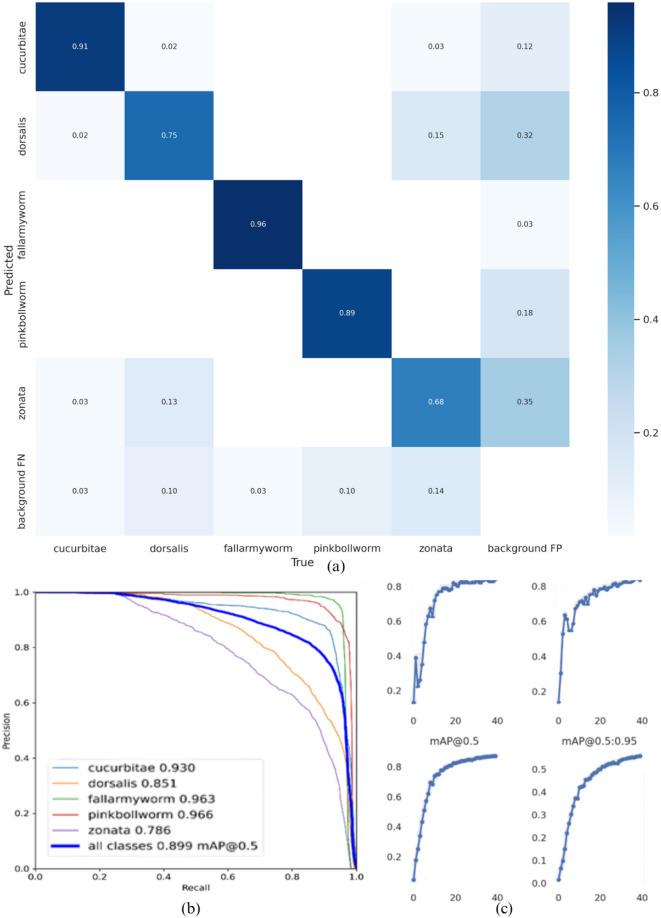
(**a**) The Confusion Matrix of Yolov7 extracted using validation dataset; (**b**) Precision-Recall curve of YOLOv7; **c**) Key performance matrix including Precision Recall, mAP@50 and mAP@50:95 of YOLOv7 model.

**Fig. 5:**
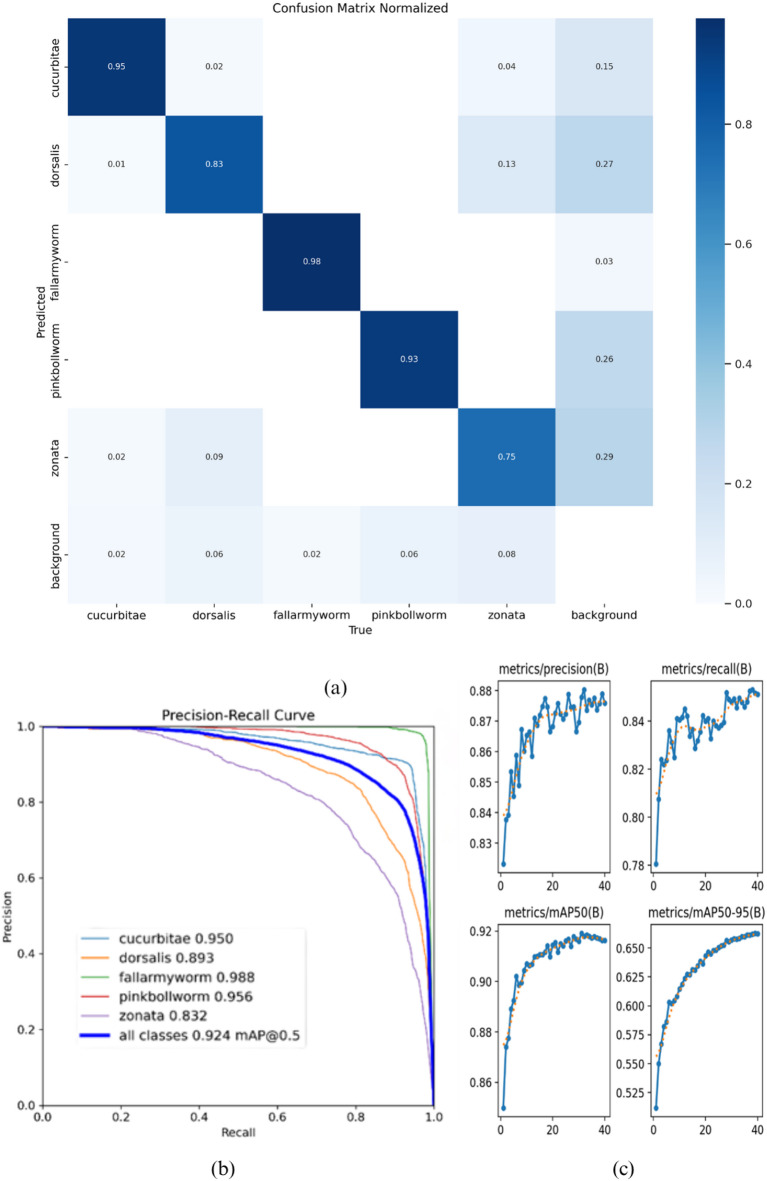
(**a**) The Confusion Matrix of Yolov8 extracted using validation dataset; (**b**) Precision-Recall curve of YOLOv8; **c**) Key performance matrix including Precision Recall, mAP@50 and mAP@50:95 of YOLOv8 model.

**Fig. 6:**
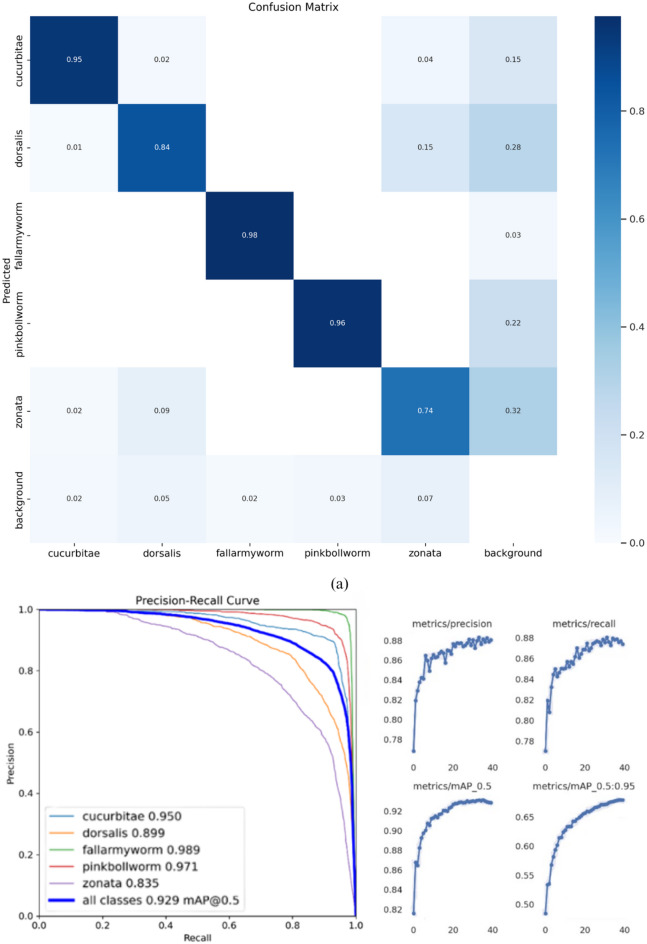
(**a**) The Confusion Matrix of Yolov9 extracted using validation dataset; (**b**) Precision-Recall curve of YOLOv9; **c**) Key performance matrix including Precision Recall, mAP@50 and mAP@50:95 of YOLOv9 model.

**Fig. 7:**
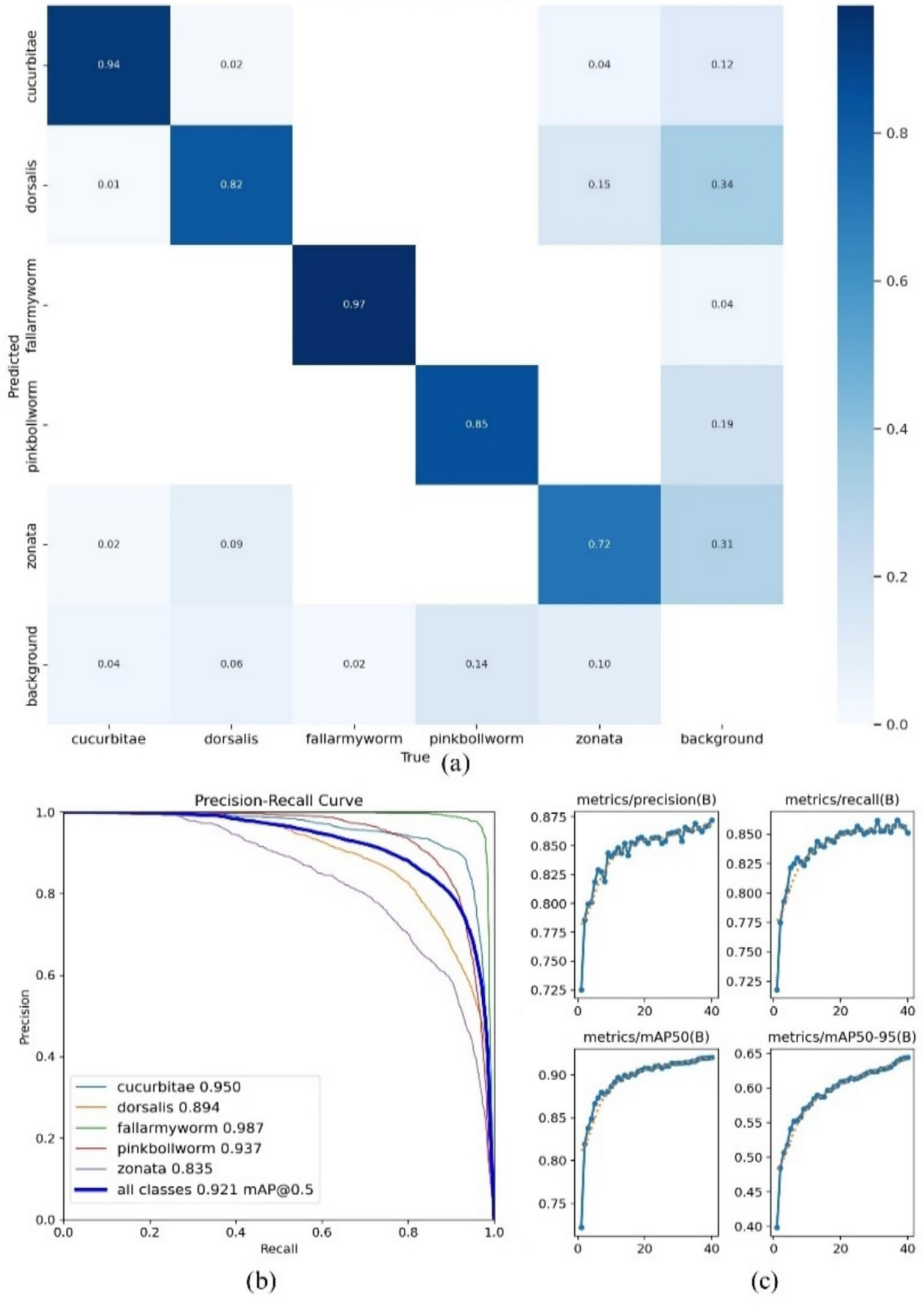
(**a**) The Confusion Matrix of Yolov10 extracted using validation dataset; (**b**) Precision-Recall curve of YOLOv10; **c**) Key performance matrix including Precision Recall, mAP@50 and mAP@50:95 of YOLOv10 model.

**Fig. 8:**
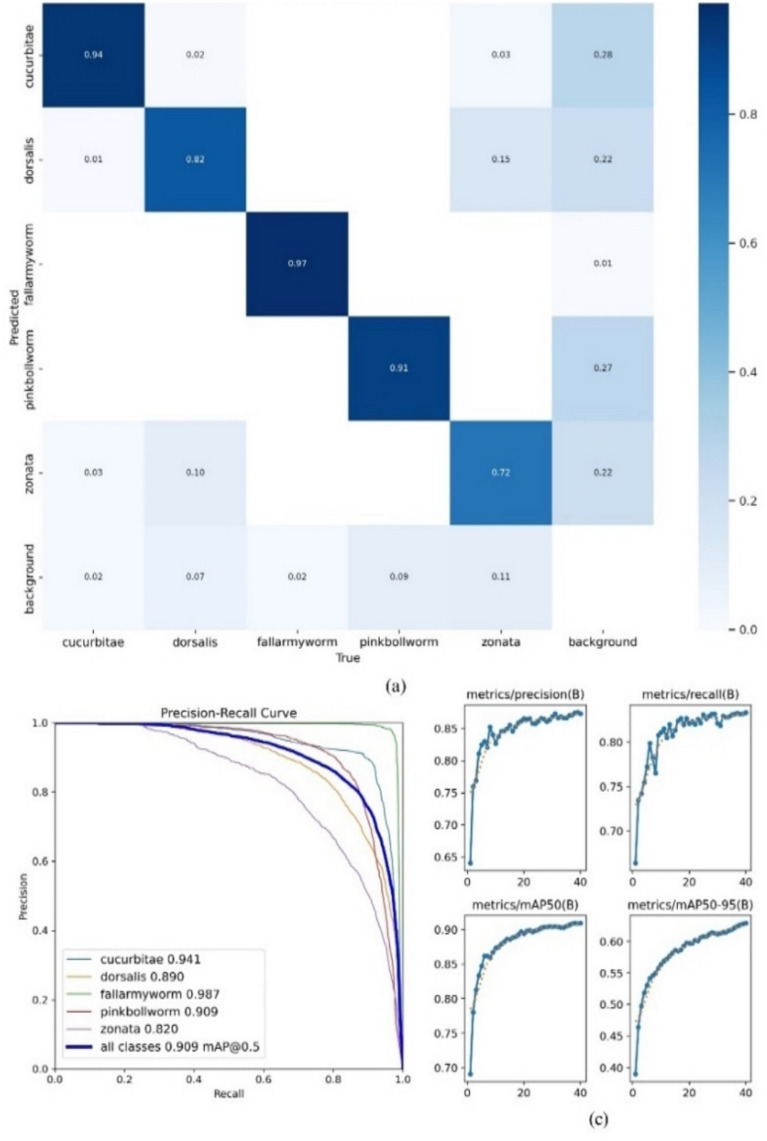
(**a**) The Confusion Matrix of Yolov11 extracted using validation dataset; (**b**) Precision-Recall curve of YOLOv11; **c**) Key performance matrix including Precision Recall, mAP@50 and mAP@50:95 of YOLOv11 model.

**Fig. 9:**
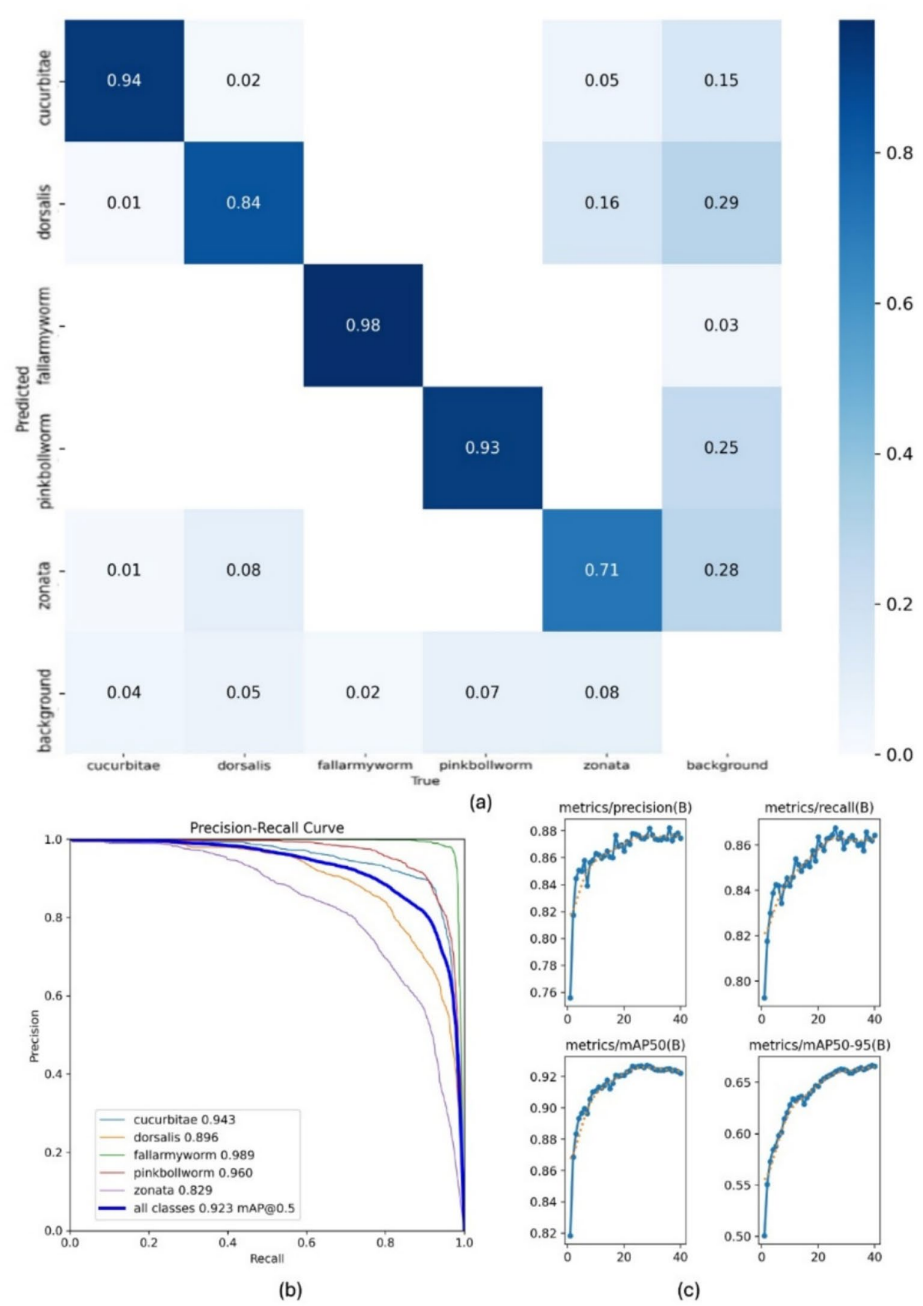
(**a**) The Confusion Matrix of Yolov12 extracted using validation dataset; (**b**) Precision-Recall curve of YOLOv12; (**c**) Key performance matrix including Precision Recall, mAP@50 and mAP@50:95 of YOLOv12 mode.

**Fig. 10:**
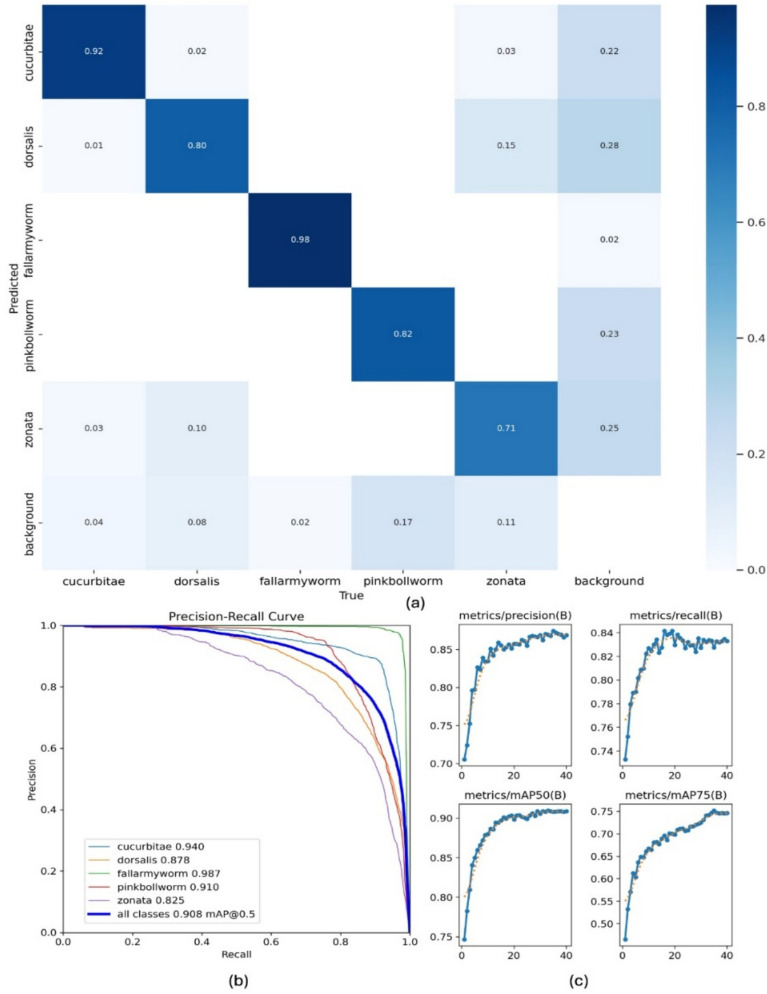
(**a**) The Confusion Matrix of Yolov13 extracted using validation dataset, (**b**) Precision-Recall curve of YOLOv13; **c**) Key performance matrix including Precision Recall, mAP@50 and mAP@50:95 of YOLOv13 model.

To evaluate the performance of the models, we conducted a comprehensive set of experiments on the validation dataset. Figure 3 (b, d, f, h, j, l, and n) presents examples of detection results from the validation dataset using YOLOv7-v13 models, respectively. The results demonstrate that YOLOv9 outperforms the other models in terms of detection accuracy under the Five-Pest dataset conditions. YOLOv7 showed relatively higher confusion between *Bactrocera zonata* and *Bactrocera dorsalis*, leading to higher false positive and false negative rates, whereas YOLOv8 and YOLOv9 demonstrated improved species separation. YOLOv12 exhibited detection behavior comparable to mid-generation models (YOLOv8-YOLOv10), maintaining stable precision and recall across most pest classes (Figs. 4, 5, 6, 7, 8, 9, 10). YOLOv13 demonstrated competitive detection capability but with slightly reduced recall consistency for visually similar fruit fly classes under dense clustering conditions (Fig. 10). All evaluated models (YOLOv7-v13) were able to classify fall-armyworm with high accuracy; however, YOLOv9 achieved the highest detection confidence among the evaluated architectures (Fig. 6). Interestingly, YOLOv7 outperformed YOLOv8 in the classification of pink bollworm, likely due to its E-ELAN architecture (Figs. 4,5). The E-ELAN design leverages multi-scale feature learning by fusing information across multiple resolutions, improving its ability to detect smaller objects. On the other hand, YOLOv8, which employs an anchor-free detection mechanism, is computationally more efficient but struggles with smaller objects, particularly in scenarios with significant overlap. In contrast, YOLOv9 utilizes the GELAN architecture, which offers greater scalability than E-ELAN but comes with a higher computational cost (Fig. 6). This scalability allows YOLOv9 to handle more complex tasks, contributing to its superior performance, especially in terms of confidence and accuracy in the detection of multiple object classes.

YOLOv9 achieved the highest mean Average Precision (mAP) of 0.929, followed by YOLOv8 (0.924), YOLOv12 (0.923), YOLOv10 (0.921), and YOLOv11 (0.909), YOLOv13 (0.908), and YOLOv7 with a mAP of 0.899 on the Five-Pest dataset (Figs. 4, 5, 6, 7, 8, 9, 10). Despite its superior accuracy, YOLOv9 exhibited higher computational demands compared to several earlier YOLO versions, particularly in terms of training time and resource usage. Figures 4, 5, 6, 7, 8, 9, 10c present key performance metrics, including Precision, Recall, mAP@0.5, and mAP@0.5:0.95 for the YOLOv7-13 models, respectively. The Precision-Recall curves for these models are shown in Figs. 4, 5, 6, 7, 8, 9, 10b, while the confusion matrices for each model are depicted in Figs. 4, 5, 6, 7, 8, 9, 10a, illustrating the models’ performance across pest classes in the Five-Pest dataset.

Regarding evaluation metrics, mAP@0.5 represents the mean Average Precision where the IoU threshold is set to 0.5. This metric provides insight into the model’s overall precision and ability to correctly detect objects with moderate overlap between predicted and ground truth bounding boxes. mAP@0.5:0.95, on the other hand, represents the mean Average Precision averaged over IoU thresholds ranging from 0.5 to 0.95 in increments of 0.05. This metric offers a more rigorous and comprehensive assessment of the model’s precision, capturing its performance at varying levels of overlap between predicted and actual bounding boxes. The confusion matrix for each model illustrates classification performance by comparing predicted labels to actual labels. The matrix is composed of True Positives (TP), False Negatives (FN), and False Positives (FP). In this context, True Positives (TP) represent correctly identified pest species. False Negatives (FN) occur when the model fails to detect an object that is present (i.e., the model predicts a negative result, but the ground truth is positive). False Positives (FP) represent cases where the model incorrectly detects an object class that is not present.

Figure 4(a) presents the confusion matrix for the YOLOv7 model, highlighting its tendency to misclassify 39% of the background (non-object instances) as *Bactrocera dorsalis*. This indicates significant confusion between the background and this specific species, leading to a high false positive rate. Furthermore, 15% of the actual *Bactrocera dorsalis* instances were incorrectly classified as *Bactrocera zonata*, reflecting the model’s difficulty distinguishing between these two closely related species. This misclassification not only increases the number of false negatives for *Bactrocera dorsalis* but also increases the false positive rate for *Bactrocera zonata* (Table 4).

**Table 4:** Validation results of YOLOv7-v9 on the Five-Pest dataset.

Class	Precision	Recall	mAP@50	Precision	Recall	mAP@50	Precision	Recall	mAP@50
	*YOLOv7 model*			*YOLOv8 model*			*YOLOv9 model*		
All	0.844	0.843	0.899	0.876	0.869	0.924	0.88	0.874	0.929
Cucurbitae	0.864	0.916	0.931	0.896	0.939	0.95	0.896	0.928	0.95
Dorsalis	0.740	0.777	0.85	0.823	0.817	0.893	0.824	0.815	0.899
Fall-armyworm	0.955	0.944	0.963	0.969	0.973	0.988	0.977	0.968	0.989
Pink-bollworm	0.911	0.926	0.965	0.907	0.885	0.956	0.919	0.935	0.971
Zonata	0.750	0.654	0.784	0.785	0.731	0.832	0.785	0.725	0.835

In comparison, the confusion matrix in Fig. 5(a) for the YOLOv8 model demonstrates a noticeable improvement in handling these misclassifications. YOLOv8 misclassified 29% of the background as *Bactrocera dorsalis*, which is a significant reduction compared to YOLOv7. This reduction in background misclassification indicates better detection performance and a lower false positive rate. Moreover, the misclassification between the two species also improved, with only 9% of *Bactrocera dorsalis* instances being wrongly classified as *Bactrocera zonata*, reflecting a better differentiation between these species. Figure 6(a) displays the confusion matrix for the YOLOv9 model, which further improves the performance of other YOLO versions. YOLOv9 misclassified 28% of the background as *Bactrocera dorsalis*, slightly better than YOLOv8 in reducing background confusion. Like YOLOv8, YOLOv9 also exhibited a low misclassification rate between the species, with 9% of *Bactrocera dorsalis* instances misclassified as *Bactrocera zonata*. In Fig. 7(a), the confusion matrix of YOLOv10 displays 34% of *Bactrocera dorsalis* as background which is lower than YOLOv9 and YOLOv8. The confusion matrix of YOLOv11 in Fig. 8(a) displays 22% of *Bactrocera dorsalis* as background, which is better than all other versions of YOLOv (7–10). However, the recall rate of YOLOv11 is 0.788, which is lower than other compared versions of YOLO except YOLOv7, with a recall rate of 0.777 for class *Bactrocera dorsalis* (Tables 4, 5, 6)*.* The consistent improvement in reducing both background misclassification and species confusion underscores the enhanced precision of YOLOv9 due to its more advanced GELAN architecture, which allows for better multi-scale feature representation and superior classification accuracy.

**Table 5:** Validation results of YOLOv10-v11 on the five-pest dataset.

Class	Precision	Recall	mAP@50	Precision	Recall	mAP@50
	YOLOv10 model			YOLOv11 model		
All	0.872	0.851	0.921	0.874	0.833	0.909
Cucurbitae	0.895	0.924	0.95	0.884	0.913	0.941
Dorsalis	0.805	0.818	0.894	0.827	0.788	0.89
Fall-armyworm	0.962	0.971	0.987	0.978	0.969	0.987
Pink-bollworm	0.916	0.825	0.937	0.871	0.827	0.909
Zonata	0.784	0.719	0.835	0.811	0.669	0.82

**Table 6:** Validation results of YOLOv12-v13 on the five-pest dataset.

Class	Precision	Recall	mAP@50	Precision	Recall	mAP@50
	YOLOv12 model			YOLOv13 model		
All	0.872	0.858	0.923	0.875	0.827	0.908
Cucurbitae	0.888	0.918	0.944	0.878	0.916	0.940
Dorsalis	0.816	0.813	0.893	0.817	0.787	0.878
Fall-armyworm	0.971	0.975	0.991	0.970	0.972	0.987
Pink-bollworm	0.880	0.878	0.952	0.910	0.779	0.910
Zonata	0.804	0.707	0.835	0.800	0.684	0.825

The confusion matrix for YOLOv12 (Fig. 9a) demonstrates strong classification performance across most pest classes, achieving correct classification rates of 0.94 for Cucurbitae, 0.84 for Dorsalis, 0.98 for fall armyworm, 0.93 for pink bollworm, and 0.71 for Zonata. Background confusion was most prominent for Dorsalis (29%) and Zonata (28%), reflecting the challenge of distinguishing fruit fly classes from complex field backgrounds. Misclassification between Dorsalis and Zonata remained moderate, with 16% of dorsalis instances misclassified as Zonata and 8% of Zonata instances misclassified as dorsalis. The confusion matrix for YOLOv13 (Fig. 10a) shows slightly reduced classification accuracy for certain classes, with correct classification rates of 0.92 for Cucurbitae, 0.80 for Dorsalis, 0.98 for fall armyworm, 0.82 for pink bollworm, and 0.71 for Zonata. Background misclassification increased for pink bollworm (17%) compared to YOLOv12, while background confusion for Dorsalis remained high at 28%. Species confusion between Dorsalis and Zonata remained comparable, with 15% of Dorsalis instances misclassified as Zonata and 10% of Zonata instances misclassified as Dorsalis.

**Fig. 11:**
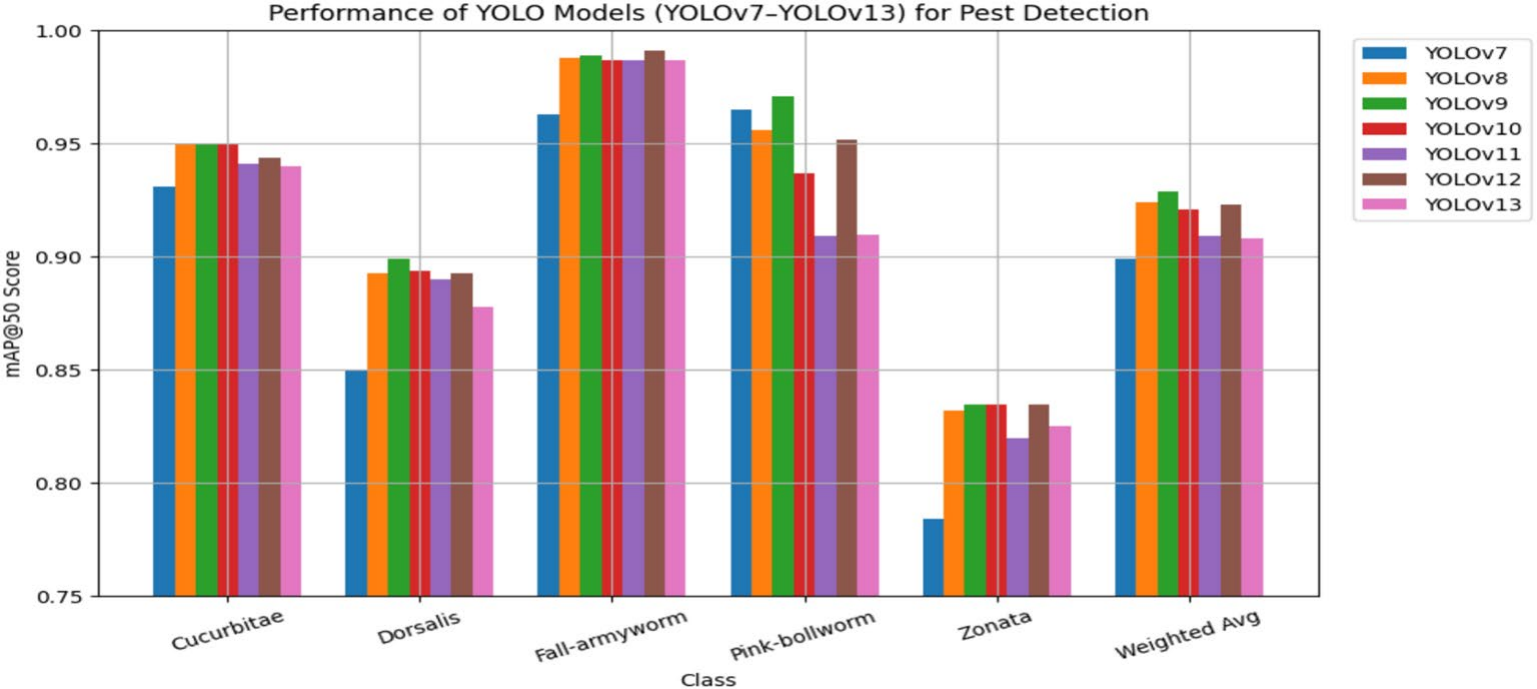
Comparison of YOLO Model Performance for Pest Detection: The histogram illustrates the mAP@50 scores of YOLOv7 to YOLOv13 across different pest classes. The weighted average highlights the overall model performance.

Table 7 presents the class-wise detection performance of the evaluated YOLO models on the Five-Pest dataset, and Fig. 11 presents the histogram illustrating the mAP@50 scores across different pest classes. The weighted average highlights the overall model performance across species. The histogram shows that YOLOv9 achieved the highest overall mAP across classes, followed by YOLOv8, YOLOv12, YOLOv10, and YOLOv11. YOLOv7 showed comparatively stronger performance for pink bollworm detection despite its relatively small size in the dataset.

**Table 7:** Class-wise mean Average Precision (mAP@50) obtained by YOLOv7-v13 models on the Five Pest Dataset. The table reports detection performance for each pest species and the weighted average mAP@50 across all classes.

Class	YOLOv7	YOLOv8	YOLOv9	YOLOv10	YOLOv11	YOLOv12	YOLOv13
Cucurbitae	0.931	0.95	0.95	0.95	0.941	0.944	0.940
Dorsalis	0.85	0.893	0.899	0.894	0.89	0.893	0.878
Fall-armyworm	0.963	0.988	0.989	0.987	0.987	0.991	0.987
Pink-bollworm	0.965	0.956	0.971	0.937	0.909	0.952	0.910
Zonata	0.784	0.832	0.835	0.835	0.82	0.835	0.825
Weighted Average	0.899	0.924	0.929	0.921	0.909	0.923	0.908

To evaluate the computational efficiency of the models, Table 8 reports the training time, inference latency, and frames-per-second (FPS) achieved by each YOLO architecture. YOLOv10 demonstrated the fastest inference performance with the lowest latency (6.5 ms per image) and the highest FPS (153.8), making it particularly suitable for real-time deployment. YOLOv8 and YOLOv11 also showed efficient inference speeds. In contrast, YOLOv7 exhibited the highest inference latency (24.5 ms per image), which may be attributed to its anchor-based detection mechanism and heavier feature aggregation design compared with later YOLO architectures that incorporate more optimized computation pipelines. Although YOLOv9 achieved the best detection accuracy, its inference speed was lower than that of YOLOv8 and YOLOv10, illustrating the trade-off between detection accuracy and computational efficiency. These results highlight that model selection for real-world pest monitoring systems should balance detection performance with inference efficiency.

**Table 8:** Computational efficiency comparison of YOLOv7-YOLOv13 models on the Five-Pest dataset. Training time (hours), inference latency (milliseconds per image), and frames per second (FPS) were measured on a Tesla T4 GPU using an input resolution of 640×640 pixels.

Model	Training Time (hours)	Inference Latency (ms/image)	FPS
YOLOv7	17.40	24.5	40.8
YOLOv8	13.67	7.8	128.2
YOLOv9	21.64	14.2	70.4
YOLOv10	9.20	6.5	153.8
YOLOv11	10.12	8.9	112.4
YOLOv12	21.10	13.8	72.5
YOLOv13	17.50	17.5	57.1

**Fig. 12:**
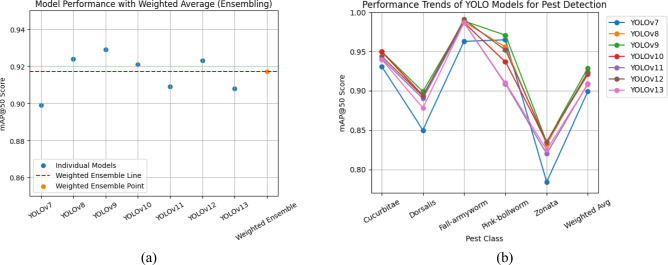
Performance Evaluation of YOLO Models with Weighted Ensemble Approach (**a**) Model-wise mAP@50 scores with a weighted ensemble average for balanced detection (**b**) Performance trends of YOLO models across pest classes, highlighting detection variations.

**Fig. 13:**
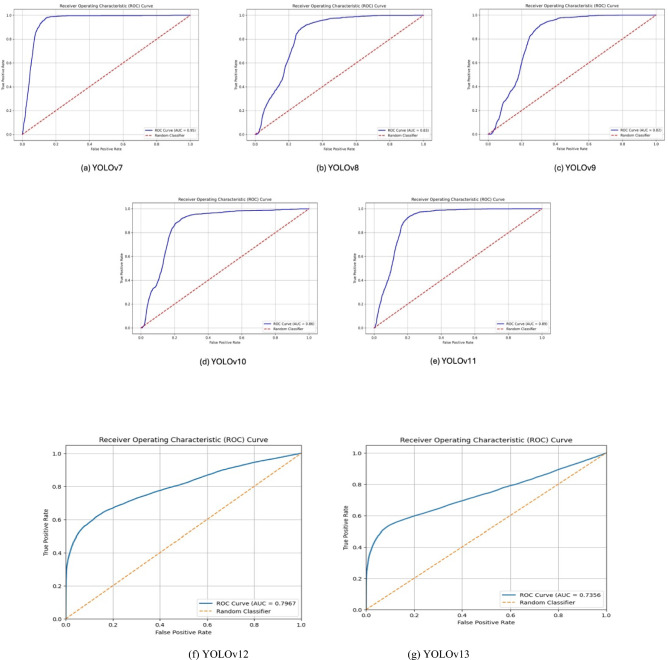
ROC curves for YOLO models on pest detection. (**a**) YOLOv7 (AUC = 0.95)—shows strong classification separability under the ROC evaluation, although its localization accuracy (mAP) is lower than several later YOLO versions (**b**) YOLOv8 (AUC = 0.83)—Moderate AUC, suggesting good classification ability but with some misclassification issues (**c**) YOLOv9 (AUC = 0.82)—Similar performance to YOLOv8, with a slightly lower AUC, indicating minor trade-offs in precision (**d**) YOLOv10 (AUC = 0.86)—Better performance than YOLOv8 and YOLOv9, demonstrating improved classification capability (**e**) YOLOv11 (AUC = 0.89)—Higher AUC than YOLOv8-YOLOv10, showing more balanced detection accuracy across pest classes (**f**) YOLOv12 (AUC = 0.79)—Slightly lower AUC compared to earlier models, indicating reduced overall discrimination stability across detection thresholds (**g**) YOLOv13 (AUC = 0.7356)—Lowest AUC among evaluated models, suggesting comparatively lower global classification separability under the Five-Pest dataset conditions.

Table 8 presents the breakdown of each model’s contribution to the weighted average, including their mAP@50 scores, assigned weights, and individual weighted contributions. The performance evaluation of YOLO models (v7-v13) with the weighted ensemble approach is demonstrated in Figs. 12 and 13. The graph (a) compares the performance of individual YOLO models (YOLOv7 to YOLOv13) based on mAP@50 scores, with the red dashed line representing the weighted ensemble average. The line plot in Fig. 12(b) illustrates the mAP@50 scores of different YOLO models across various pest classes. The performance varies among species, with all models performing best on Fall Armyworm and least Table 9 on Zonata, indicating class-specific detection challenges. The ensemble approach balances contributions from all models, ensuring a more robust detection performance across pest classes, offering more stable and generalized detection performance, especially for difficult classes such as Zonata and Dorsalis, where individual models exhibit higher variability. This reinforces the ensemble model’s value in mitigating model-specific weaknesses and enhancing robustness.

**Table 9:** Model (YOLOv7-v13) contribution to weighted average.

Model	Weighted average	Weight	Weighted contribution
YOLOv7	0.899	0.140	0.126
YOLOv8	0.924	0.144	0.133
YOLOv9	0.929	0.145	0.135
YOLOv10	0.921	0.144	0.132
YOLOv11	0.909	0.142	0.129
YOLOv12	0.922	0.144	0.133
YOLOv13	0.909	0.142	0.129

**Fig. 14:**
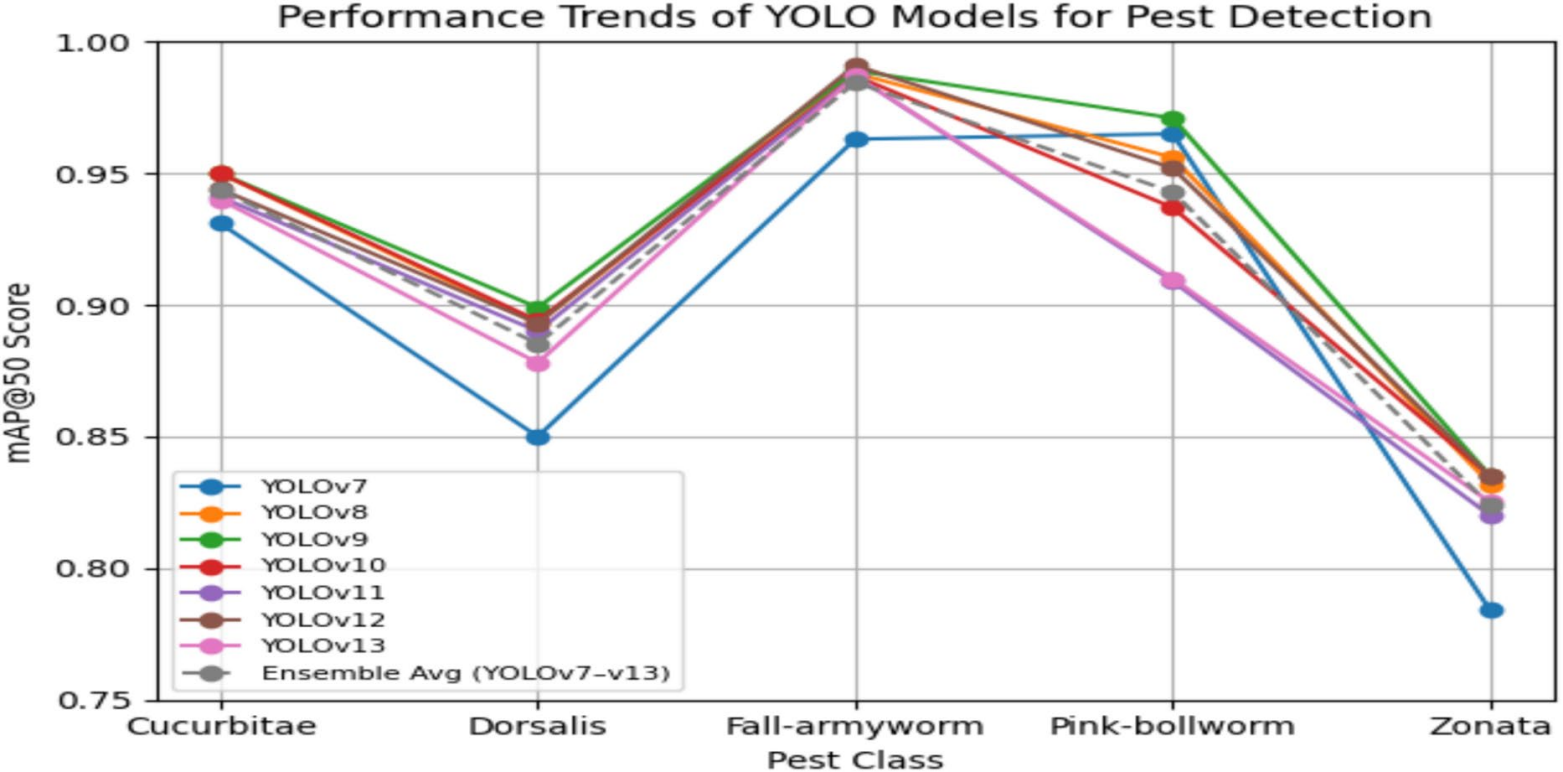
Comparative performance of YOLOv7-YOLOv13 models and the weighted ensemble approach on the Five-Pest dataset.

Figure 14 illustrates the comparative performance of the evaluated YOLO models together with the weighted ensemble approach. The ensemble method provides a more stable performance across pest classes by combining the strengths of individual models. While YOLOv9 achieved the highest individual mAP@50 score, the ensemble approach improves consistency across difficult classes such as Zonata and Dorsalis. This reinforces the ensemble model’s value in mitigating model-specific weaknesses and enhancing robustness. However, despite its improved precision, the ensemble model is not suited for deployment in real-time or edge-based applications like smart traps, due to the computational overhead of running multiple models concurrently. Therefore, it serves primarily as an upper-bound benchmark and a potential candidate for model distillation or offline analysis, rather than a directly deployable solution in resource-constrained environments.

## Discussion

This study presents a comparative evaluation of state-of-the-art YOLO-based object detection models (YOLOv7-v13) on the novel Five-Pest dataset comprising images of five economically significant insect pests: three fruit- fly species, pink bollworm, and fall armyworm. The primary goal was to assess model performance across multiple dimensions, including training efficiency, detection accuracy (mAP@50), recall, and robustness via ROC-AUC, under a standardized training environment using identical hyperparameters and augmentation protocols on a Tesla T4 GPU. In the context of this study, the deployment environment is a low-power edge device integrated into the smart traps’ hardware, designed for real-time pest detection in remote agricultural fields with limited connectivity. Unlike GPU-enabled cloud servers, edge devices have strict constraints on memory, power, and processing capacity, necessitating the selection of lightweight models for practical use. While YOLOv9 and YOLOv10 demonstrate higher mAP scores, their computational demands may exceed the capabilities of typical edge hardware unless optimized through pruning or quantization. Similarly, YOLOv12 and YOLOv13, while offering competitive detection performance and improved feature representation stability, introduce additional computational complexity due to deeper feature processing and expanded parameterization, which may further limit direct deployment on resource-constrained edge devices without model compression or hardware acceleration. The ensemble approach evaluated in this study improves detection consistency across pest classes; however, it increases inference cost approximately proportional to the number of combined models, making it more suitable as an upper-bound performance reference or for offline analysis rather than real-time edge deployment. In this work, ‘real-time’ refers to autonomous pest identification with minimal latency (≤ 2 s per image) and a practical capture frequency (1–2 images per hour). This enables aggregated pest counts over 6–12 h windows, aligning with daily farm management decisions. The trade-off between accuracy and deploy ability is therefore critical when selecting object detection models for edge-based agricultural monitoring systems like smart traps.

The evaluation results reveal important insights into model performance and suitability for edge deployment. YOLOv9 achieved the highest mAP@50 (92.9%), outperforming YOLOv8 (92.4%) and other variants. However, YOLOv8 completed training nearly 8 h faster than YOLOv9, highlighting its architectural efficiency. Despite YOLOv10 (92.1%) and YOLOv11 (90.9%) showing slightly lower precision, YOLOv11 demonstrated a favorable balance between accuracy and classification confidence, making it a viable candidate for real-world use on constrained hardware. YOLOv12 (92.2%) achieved performance comparable to YOLOv8 and YOLOv10 but required substantially longer training time. In contrast, YOLOv13 (90.9%) exhibited the highest computational cost among all evaluated variants in this study, requiring the longest training time, indicating that while it may provide architectural improvements, it is currently less suitable for resource-constrained or edge-based agricultural deployment scenarios. While it demonstrates competitive detection capability, its computational overhead currently limits its suitability for resource-constrained or edge-based agricultural deployment scenarios.

Interestingly, ROC-AUC analysis (Fig. 13) shows that YOLOv7 attains the highest overall AUC of 0.95, indicating its strong ability to discriminate between pest and background across varying confidence thresholds, particularly beneficial for species with highly similar appearance. In contrast, YOLOv8 and YOLOv9, while accurate, suffered from lower AUC values, suggesting elevated false positive rates. YOLOv12 demonstrated ROC performance comparable to mid-generation models (YOLOv8-YOLOv10), indicating stable classification confidence despite increased architectural complexity. YOLOv13 exhibited ROC behavior similar to YOLOv11 in our experiments, suggesting that while architectural depth increased, classification separability did not proportionally improve under the Five-Pest dataset conditions. For pest classes like *Bactrocera dorsalis* and *Bactrocera zonata*, this implies a need for enhanced feature engineering to resolve class confusion. These findings support previous work by^21^^,^^19^^,^^29^, who emphasized the trade-offs between accuracy, efficiency, and deployment context.

For our targeted application, precise localization and counting of individual pests to inform localized interventions, mAP@50 is the key performance metric, since it measures how accurately predicted boxes overlap ground truth at a fixed IoU threshold. While AUC (with YOLOv7 at 0.95) reflects classification confidence across all thresholds, it does not guarantee high localization precision under operational settings. Therefore, YOLOv9’s superior mAP@50 (0.929) makes it the preferred model for field deployment, whereas YOLOv7’s higher AUC could guide use cases that emphasize threshold-agnostic detection confidence (e.g., real-time alert systems). By integrating these advanced YOLO variants into AI-powered smart traps, precision pest management becomes feasible, reducing reliance on blanket pesticide use. This aligns with the environmental and cost-saving benefits noted by Alves et al*.* (2024), reinforcing the potential of such systems in scalable, sustainable agriculture.

While the YOLO variants demonstrated strong detection performance, particularly YOLOv9 in precision, YOLOv8 in training efficiency, and YOLOv7 in detection confidence, challenges remain under complex field conditions. Tailored augmentations and Pest-Guided Interpolation (PGI) enhanced robustness to lighting and background variability, yet detection failures under dense occlusion and cluttered environments remain problematic. This highlights the need for sensor fusion, e.g., incorporating thermal or depth imaging, to enhance reliability. Furthermore, models trained on RGB images showed limited generalizability in adverse conditions (low light, glare). Exploring alternative architectures such as Vision Transformers, Deformable DETR, or hybrid CNN-transformers may yield gains in detection fidelity. However, high computational demands of two-stage detectors (e.g., Faster R-CNN) constrain their use in edge devices, pointing to lightweight yet accurate architectures as the optimal path forward [40,42].

Moreover, the increasing computational requirements observed in later YOLO variants (particularly YOLOv12 and YOLOv13) emphasize the importance for model optimization via pruning, quantization, and transfer learning to ensure scalable deploymen [44 ]. Dataset limitations, restricted to five pest species, also raise concerns about ecological representativeness. Excluding other pests and beneficial insects could skew decision-making. Overfitting risks are elevated due to class imbalance and repeated backgrounds. To improve generalization, future research should expand the dataset across pests, regions, and field conditions while incorporating continual learning and domain adaptation.

To further enhance detection performance, we evaluated an ensemble model combining YOLOv7 to YOLOv13 via prediction averaging. As shown in Fig. 12, the ensemble achieved more consistent accuracy across all pest classes, particularly improving detection of challenging species like *Zonata* and *Dorsalis*. By leveraging complementary strengths of individual models, the ensemble reduced variance and improved generalizability, making it a strong candidate for offline analysis or centralized decision support. However, its high computational cost limits feasibility for real-time, edge-based deployment. Future work could explore knowledge distillation or lightweight surrogate models to approximate ensemble performance in resource-constrained settings. Additionally, embedding pest detection in a broader IoT ecosystem, combining soil sensors, weather data, and automated actuation, could enable holistic crop management and sustainable smart farming.

## Conclusion

This study provides a comprehensive evaluation of seven YOLO-based object detection models (YOLOv7-YOLOv13) for automated pest identification using the novel Five-Pest dataset, targeting critical insect threats to agriculture. The results highlight a clear trade-off: YOLOv9 offers the highest precision (mAP@50 = 92.9%) and is most suitable for applications where detection accuracy is paramount. In contrast, YOLOv8 demonstrates the fastest training and efficient inference, making it well-suited for real-time deployment on edge devices in resource-constrained agricultural environments. YOLOv7, while not leading in mAP, achieves the highest Area Under Curve (AUC), score indicating strong classification confidence, useful in applications prioritizing reliable alert systems over precise localization. YOLOv12 achieved performance comparable to YOLOv8 and YOLOv10 while maintaining stable precision and recall characteristics but required longer training time. In contrast, YOLOv13 demonstrated competitive detection capability but incurred the highest computational cost among evaluated models, limiting its practicality for edge-based agricultural deployment under current configurations. A multi-member model ensemble built from YOLOv7-v13 further improved per-class consistency and addressed misclassification in difficult pest classes. However, its computational expense renders it impractical for edge-based systems, suggesting a role in offline analysis or as a teacher model for lightweight alternatives via knowledge distillation. These insights emphasize the need to align model choice with the operational context in precision agriculture. Future work should focus on enhancing generalizability to new field conditions, expanding the dataset to include diverse pest species and environments, and integrating multimodal sensing for robust, scalable deployment. Smart trap systems leveraging these optimized models can play a pivotal role in reducing pesticide misuse, improving crop yields, and supporting sustainable and climate-resilient farming practices.

## Supplementary Information

Supplementary Information.

## Data Availability

A sample of the dataset is made publicly available at: https://data.mendeley.com/datasets/hgz2n5jxhp/1. The complete dataset used in this research can, however, not yet be made publicly available, as it is also still being utilized in an ongoing study. Data will be made available on demands from the corresponding and first authors.
