# Supplementary material for: Evaluation of YOLOv7-v13 models for multi-class small insect pest detection using the five-pest dataset
Source: Sci Rep. 2026 May 20;16:23059. doi: 10.1038/s41598-026-52108-3 (PMC13392103; doi:10.1038/s41598-026-52108-3)
Supplement: Supplementary file 1 — Supplementary Information. [file 41598_2026_52108_MOESM1_ESM.docx]

## **Evaluation of YOLOv7-v11 models for Multi-Class Small Insect Pest Detection Using the Five-Pest Dataset**

Ayesha Hakim^1,2^, M. Habib Ur-Rahman^3*^, Ali Hamza^2^, Munir P. Hoffmann^3^, Vakhtang Shelia^4^, Muhammad Owais^2^, Nimra Khan^2^, Muhammad Saim Ibtesam^5^, Muhammad Rashid^2^, Reimund P. Rötter^3,6^

Ayesha Hakim^1,2+^, M. Habib Ur-Rahman^+3*^, Ali Hamza^2^, Munir P. Hoffmann^3^, Vakhtang Shelia^4^, Muhammad Owais^2^, Nimra Khan^2^, Muhammad Saim Ibtesam^5^, Muhammad Rashid^2^, Reimund P. Rötter^3,6^

^1^School of Electrical Engineering and Computer Science (SEECS), National University of Sciences and Technology (NUST), Islamabad, Pakistan

^2^Institute of Computing, MNS University of Agriculture, Multan, Pakistan

^3^Tropical Plant Production and Agricultural Systems Modelling (TROPAGS), Georg-August-Universität Göttingen, Grisebachstraße 6, 37077 Göttingen, Germany

^4^Department of Agricultural and Biological Engineering, University of Florida, Gainesville, FL 32611, USA

^5^Institute of Plant Protection, MNS University of Agriculture, Multan, Pakistan

^6^Campus Centre for Biodiversity and Sustainable Land Use (CBL), Georg-August-Universität Göttingen, Büsgenweg 1, 37077 Göttingen, Germany

+ Considered as first co-author

^*^Corresponding authors: MHUR and AH ([habib.rahman@uni-goettingen.de](mailto:habib.rahman@uni-goettingen.de); ayesha.hakim@seecs.edu.pk)

**SUPPLEMANTARY MATERIAL**

### **Data Augmentation**

| 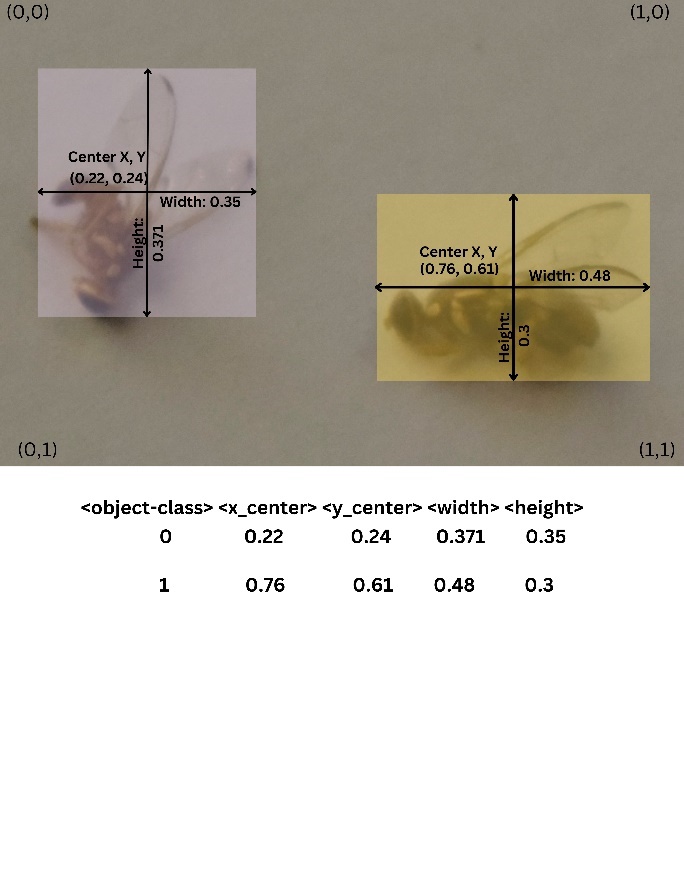 |
| --- |
| **Sup. Fig. 1**: Bounding box measurement in YOLO Darknet txt labeling format |

**2.3. Real-time Object Detection Models (Extended)**

### **YOLOv7** is a real-time object detection model released in July 2022 that belongs to the YOLO group of models (Wang *et al.,* 2023). It achieved improved accuracy and inference speed were compared with earlier object detection models. YOLOv7 utilizes a convolutional neural network (CNN) architecture with a Darknet-based backbone for feature extraction. The overall architecture consists of three main components: a backbone for extracting hierarchical image features, a neck for aggregating multi-scale features, and detection heads for predicting bounding boxes and class probabilities. Yolov7 is capable of detecting objects of different sizes using a Feature Pyramid Network (FPN) (Terven *et al.,* 2023). The FPN constructs a hierarchy of feature maps with different spatial resolutions, where each level represents features at a different scale. This multi-scale representation enables YOLOv7 to effectively detect objects of varying sizes.

The architecture of YOLOv7 is a concatenation-based design, where standard scaling techniques like depth scaling may alter the ratio between the input and output channels of a transition layer. This change aims to reduce the hardware usage of the model. YOLOv7 introduced a novel scaling strategy for concatenation-based models, ensuring that both the depth and width of the blocks are scaled by the same factor to preserve the optimal structure of the model. In YOLOv7 (Lou *et al.,* 2023) model, anchor boxes are pivotal for object detection. These predetermined bounding boxes of various shapes and sizes are positioned across the image, allowing the algorithm to predict object locations relative to these anchors. Each anchor box is associated with the specific aspect ratios and scales, further assisting the model in effectively detecting objects of different sizes. Following prediction, YOLOv7 employs Non-Maximum Suppression (NMS) to refine the bounding box outputs. NMS is a post-processing technique that compares the confidence scores of predicted boxes, retaining only the box with the highest confidence while suppressing overlapping boxes with lower scores. This process ensures accurate detection by eliminating redundant and overlapping predictions, yielding a refined set of bounding boxes for detected objects. Figures 5(a) and 5(b) show the comparison of two images obtained before and after NMS. The IOU-Threshold was deliberately set to 0.1 to demonstrate the critical role of the NMS in the post-processing technique. Using such a low threshold, even minimal overlap between bounding boxes (as low as 10%) triggers the suppression of redundant detections. This emphasizes the precision and effectiveness of NMS in ensuring that each detection represents a distinct instance, thus providing a clearer and more accurate output.

| 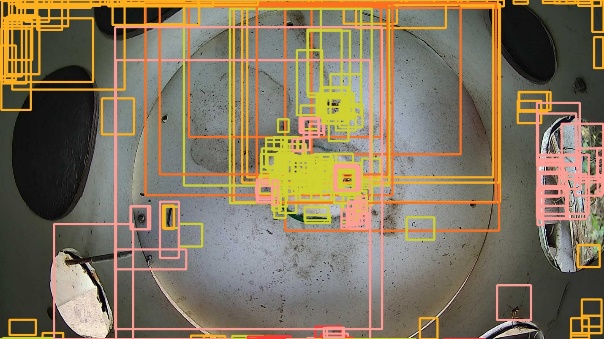 | 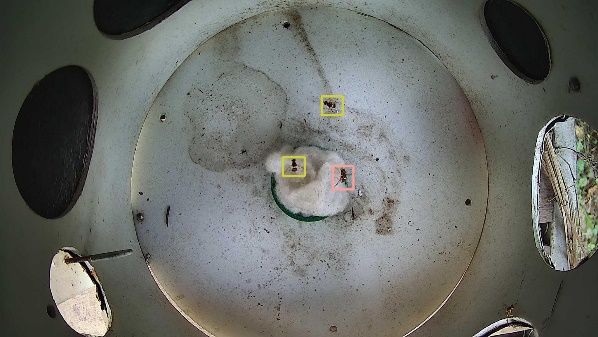 |
| --- | --- |
| (a) | (b) |
| ***Sup. Fig. 2****: Non-maximum Suppression (a) Typical output of YOLO detection containing multiple overlapping anchor boxes (b) Output after applying NMS filtering to remove redundant overlapping detection.* | |

**YOLOv8** (Lou *et al.,* 2023) was released in January 2023 by Ultralytics (Jocher *et al.,* 2023), the same developers as that of YOLOv5. YOLOv8 uses a similar backbone as YOLOv5 but differs in the C2f (Cross-stage partial bottleneck with two convolutions) module, which merges high-level features with contextual information to enhance detection accuracy. Utilizing a decoupled head, the YOLOv8 model independently handles objectness, classification, and regression, allowing each branch to focus on its specific role, thereby enhancing detection accuracy. Object classification relies on the SoftMax function that assigns probabilities to different classes. YOLOv8 employs advanced techniques such as Complete Intersection over Union (CIoU) (Zheng *et al.,* 2020) and Distribution Focal Loss (DFL) (Li *et al.,* 2020) for bounding box loss computation coupled with binary cross-entropy for classification loss calculation. This approach leads to improved object detection, specifically for smaller objects. YOLOv8 achieved state-of-the-art performance on various object detection benchmarks while maintaining high speed and efficiency. It offers both command-line interface (CLI) execution and a PIP package, making it easy for developers to use.

**YOLOv9** (Wang et al., 2024) was released in February 2024 by the same developers behind YOLOv7. The focus of YOLOv9 is to determine the most suitable objective function to minimize information loss. Additionally, it introduces a lightweight architecture - Generalized Efficient Layer Aggregation Network (GELAN) and programmable gradient information (PGI), utilizing conventional convolution operators to enhance model efficiency and speed. PGI enhances the model’s adaptability as gradient-related parameters, such as learning rate and optimization strategies, can be adjusted during training.

**YOLOv10**, released in May 2024, introduces a novel dual-label assignment strategy to enhance object detection accuracy and consistency. The model’s backbone processes input images to extract features, which are then refined and propagated through the Path Aggregation Network (PAN). This feature fusion process ensures effective detection across multiple object scales and complexities. The architecture incorporates two distinct prediction heads: one-to-many heads and one-to-one heads. The one-to-many head uses regression and classification branches to handle generalized predictions, accommodating multiple object proposals for each anchor point. Conversely, the one-to-one head is designed for precise object matching, enabling a one-to-one correspondence between predicted boxes and ground truth labels. This dual-head design ensures a balanced approach to both high recall and precision. A key innovation in YOLOv10 is the introduction of a consistent matching metric, defined in Equation 2:

| $m=s. p^{a}. IoU(b,{\hat{b})}^{\beta}$ | (2) |
| --- | --- |

Here, “s” represents the confidence score, p denotes the class probability, α and β are scaling parameters, and $IoU(b,{\hat{b})}^{\beta}$ measures the overlap between the predicted and ground truth bounding boxes. This metric plays a crucial role in maintaining prediction consistency by prioritizing high-confidence and well-aligned predictions. The backbone and PAN are presented as the feature extraction pipeline. The central section illustrates the dual heads, responsible for regression and classification tasks. The consistent matching metric is depicted, highlighting the mechanism by which the model evaluates overlapping predictions to assign precise labels. This architecture is designed to enhance adaptability in complex object detection scenarios, establishing it as a robust solution for contemporary applications.

**YOLOv11**, released in September 2024, features an advanced architecture designed to enhance efficiency and precision in object detection. YOLOv11 comprises three main sections: Backbone, the Neck, and the Detection Heads. The Backbone is responsible for feature extraction, leveraging multiple convolutional layers (Conv) and C3K2 modules to process the input image and extract both low-level and high-level features. These features are refined through the SPPF (Spatial Pyramid Pooling - Fast) module, which efficiently aggregates multi-scale features, and the C2PSA module, which applies spatial attention to focus on critical regions, thereby improving feature representation. In the Neck section, up-sampling layers and concatenation (Concat) operations are utilized to fuse features from different scales, enabling effective multi-scale feature integration. This ensures that both fine-grained and global information is preserved for subsequent processing. Finally, the Detection Heads predict objects at three scales: small, medium, and large. Each detection head processes scale-specific feature maps, ensuring robustness and accuracy across diverse object sizes. The modular design integrates innovative components like the SPPF and C2PSA modules, enhancing the model's detection precision and efficiency while maintaining the real-time performance characteristic of YOLO models.

**YOLOv12**, introduced in late 2024 as an extension of YOLO series, represents a further evolution of the YOLO family, focusing on improving detection accuracy and computational efficiency for real-time object detection tasks. The architecture follows the conventional three-stage design consisting of a backbone, neck, and detection heads. The backbone is responsible for hierarchical feature extraction and incorporates optimized convolutional blocks to capture both low-level spatial features and high-level semantic information. To enhance feature aggregation, YOLOv12 utilizes an improved feature fusion strategy in the neck, enabling better integration of multi-scale features from different layers of the backbone. This design improves the model’s capability to detect small and densely distributed objects, which are common in agricultural pest monitoring scenarios. In addition, YOLOv12 incorporates refined loss functions and improved label assignment strategies to stabilize training and improve bounding box localization accuracy. These enhancements allow YOLOv12 to achieve a better balance between detection accuracy and inference speed, making it suitable for real-time deployment in edge-based monitoring systems.

**YOLOv13**, proposed in early 2025 as a further development within the YOLO family, emphasizing improved detection robustness, model scalability, and efficient inference. Like its predecessors, YOLOv13 maintains the backbone-neck-head architecture but introduces further refinements in feature extraction and prediction mechanisms. The backbone integrates advanced convolutional modules and attention-based feature enhancement techniques to improve representation learning, particularly for small or visually similar objects. In the neck, enhanced feature pyramid structures are employed to facilitate efficient multi-scale feature fusion, allowing the model to better capture spatial relationships across different object sizes. YOLOv13 also incorporates optimized detection heads and improved training strategies to enhance classification confidence and bounding box regression accuracy. These architectural refinements enable YOLOv13 to achieve higher detection precision while maintaining real-time inference capability, making it well suited for complex object detection tasks such as multi-class insect pest monitoring in field environments.

- 1. **Model Training**


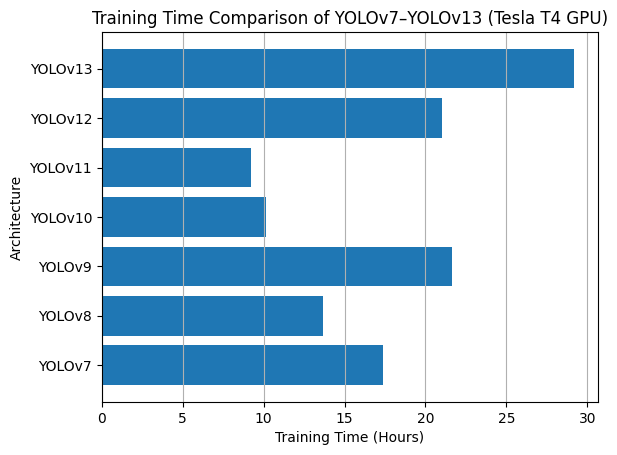


***Sup. Fig. 3****: Training time (hours) of different YOLO pest models (v7-v13) for Five Pest dataset using Tesla T4 GPU*

| 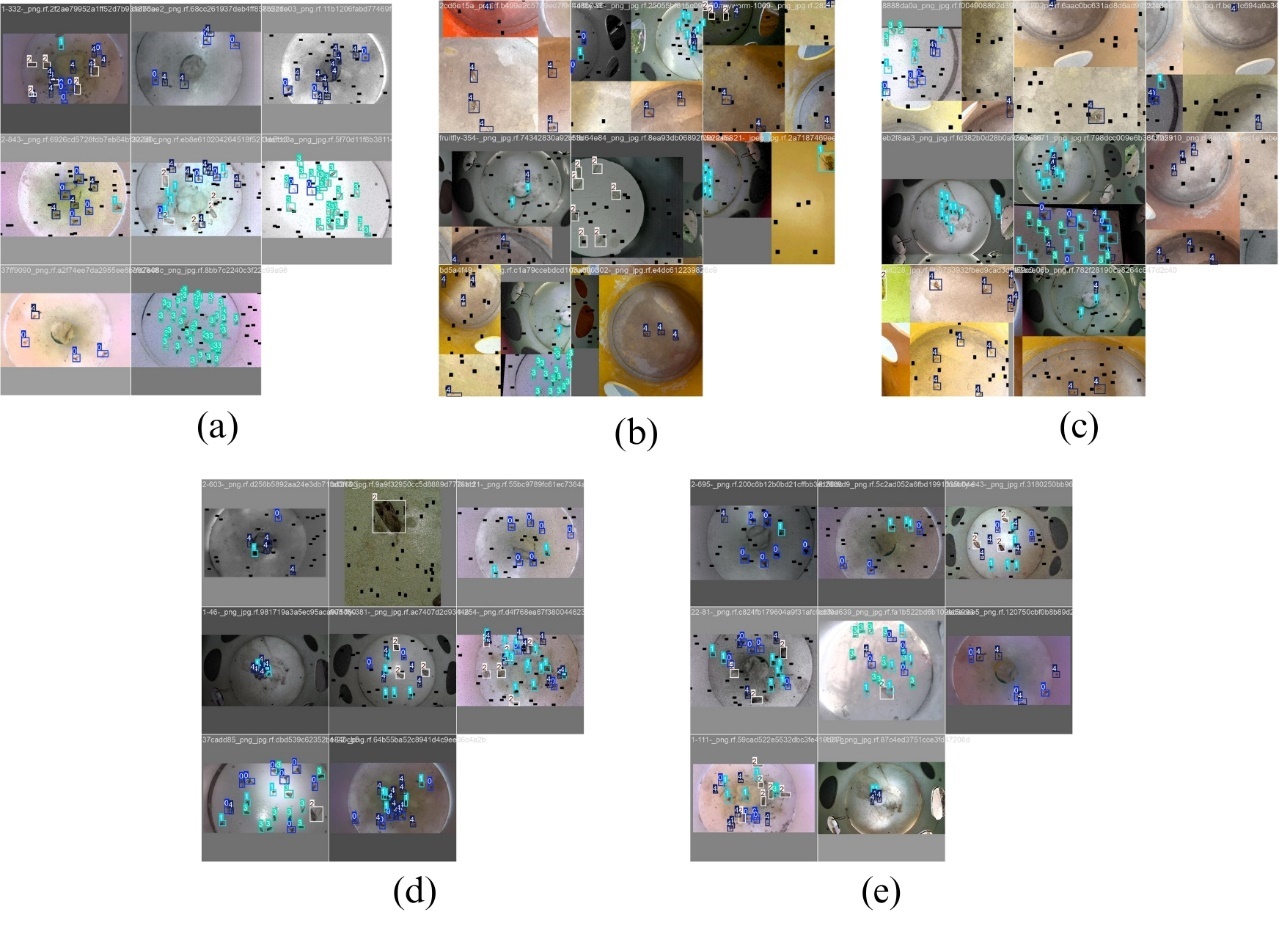 | |
| --- | --- |
| 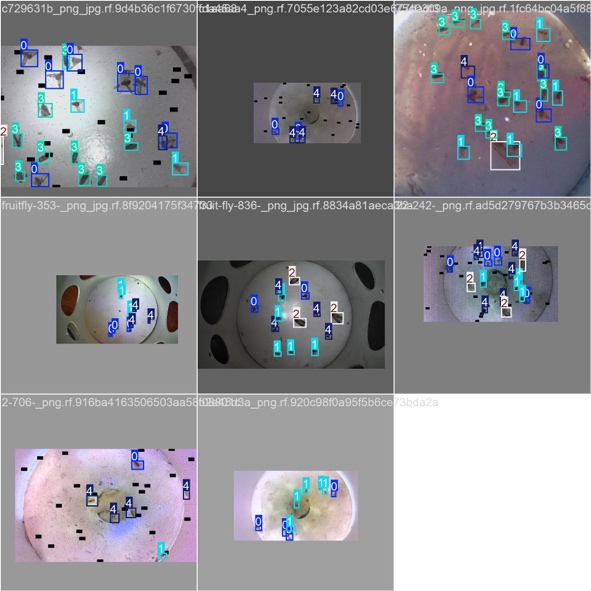 | 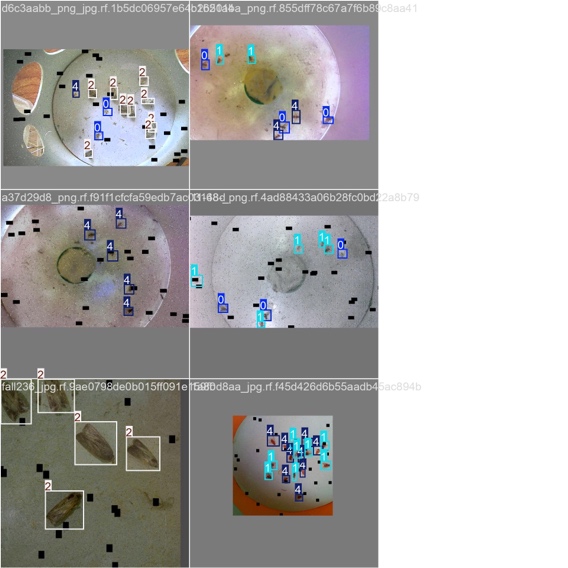 |
| (f) | (g) |
| ***Sup. Fig. 4****: Output image of each batch during model training. a) YOLOv7, b) YOLOv8, c) YOLOv9, d) YOLOv10, e) YOLOv11, f) YOLOv12, and g) YOLOv13 models* | |
